# G9a/DNMT1 co-targeting inhibits non-small cell lung cancer growth and reprograms tumor cells to respond to cancer-drugs through SCARA5 and AOX1

**DOI:** 10.1038/s41419-024-07156-w

**Published:** 2024-11-02

**Authors:** Francisco Exposito, Miriam Redrado, Diego Serrano, Silvia Calabuig-Fariñas, Aida Bao-Caamano, Sandra Gallach, Eloisa Jantus-Lewintre, Angel Diaz-Lagares, Aitor Rodriguez-Casanova, Juan Sandoval, Edurne San Jose-Eneriz, Javier Garcia, Esther Redin, Yaiza Senent, Sergio Leon, Ruben Pio, Rafael Lopez, Julen Oyarzabal, Antonio Pineda-Lucena, Xabier Agirre, Luis M. Montuenga, Felipe Prosper, Alfonso Calvo

**Affiliations:** 1 Program in Solid Tumors, Cima-Universidad de Navarra, Cancer Center Clinica Universidad de Navarra (CCUN), Pamplona, Spain; 2 CIBERONC, ISCIII, Madrid, Spain; 3 IDISNA, Pamplona, Spain; 4 Department of Pathology, Anatomy and Physiology, School of Medicine, University of Navarra, Pamplona, Spain; 5 Molecular Oncology Laboratory, Fundación Hospital General Universitario de Valencia, 46014 Valencia, Spain; 6 TRIAL Mixed Unit, Centro de Investigación Príncipe Felipe-Fundación para la Investigación del Hospital General Universitario de Valencia, 46014 Valencia, Spain; 7 Department of Pathology, Universitat de València, 46010 Valencia, Spain; 8 Epigenomics Units, Cancer Epigenomics, Translational Medical Oncology Group (ONCOGAL), Health Research Institute of Santiago de Compostela (IDIS), and Department of Clinical Analysis, University Hospital Complex of Santiago de Compostela (CHUS), Roche-CHUS Joint Unit (ONCOMET), Health Research Institute of Santiago (IDIS), 15706, Santiago de Compostela, Spain, 15706 Santiago de Compostela, Spain; 9 Department of Biotechnology, Universitat Politècnica de València, 46022 Valencia, Spain; 10 Biomarkers and Precision Medicine (UBMP) and Epigenomics Unit, IIS, La Fe, 46026 Valencia, Spain; 11 Division of Hemato-Oncology, Cima-Universidad de Navarra, Cancer Center Clinica Universidad de Navarra (CCUN), Pamplona, Spain; 12 Department of Biochemistry and Genetics, School of Sciences, University of Navarra, Pamplona, Spain; 13 Molecular Therapeutics Program, CIMA, CCUN, University of Navarra, Pamplona, Spain; 14 Hematology and Cell Therapy Service, Cancer Center Clinica Universidad de Navarra (CCUN), Pamplona, Spain; 15 Present Address: Yale Cancer Center, New Haven, CT USA

**Keywords:** Cancer, Cell biology

## Abstract

The treatment of non-small cell lung cancer (NSCLC) patients has significantly improved with recent therapeutic strategies; however, many patients still do not benefit from them. As a result, new treatment approaches are urgently needed. In this study, we evaluated the antitumor efficacy of co-targeting G9a and DNMT1 enzymes and its potential as a cancer drug sensitizer. We observed co-expression and overexpression of G9a and DNMT1 in NSCLC, which were associated with poor prognosis. Co-targeting G9a/DNMT1 with the drug CM-272 reduced proliferation and induced cell death in a panel of human and murine NSCLC cell lines. Additionally, the transcriptomes of these cells were reprogrammed to become highly responsive to chemotherapy (cisplatin), targeted therapy (trametinib), and epigenetic therapy (vorinostat). In vivo, CM-272 reduced tumor volume in human and murine cell-derived cancer models, and this effect was synergistically enhanced by cisplatin. The expression of SCARA5 and AOX1 was induced by CM-272, and both proteins were found to be essential for the antiproliferative response, as gene silencing decreased cytotoxicity. Furthermore, the expression of SCARA5 and AOX1 was positively correlated with each other and inversely correlated with G9a and DNMT1 expression in NSCLC patients. SCARA5 and AOX1 DNA promoters were hypermethylated in NSCLC, and SCARA5 methylation was identified as an epigenetic biomarker in tumors and liquid biopsies from NSCLC patients. Thus, we demonstrate that co-targeting G9a/DNMT1 is a promising strategy to enhance the efficacy of cancer drugs, and SCARA5 methylation could serve as a non-invasive biomarker to monitor tumor progression.

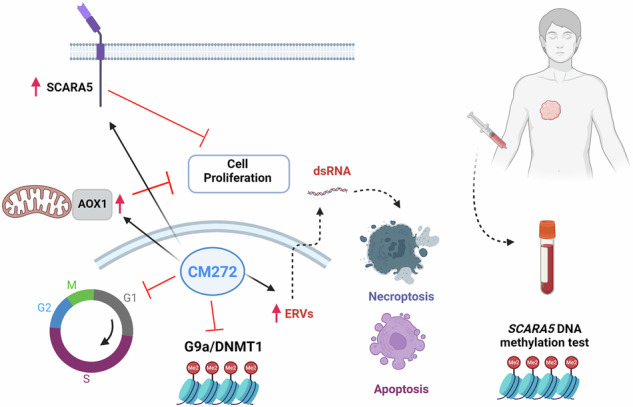

## Introduction

Lung cancer remains as the leading cause of cancer-related mortality in both men and women [1]. Despite advances in chemotherapy, targeted therapy and immunotherapy, the average 5-year survival for patients with advanced NSCLC is ~28%. Non-small cell lung cancer (NSCLC) represents 85% of all lung cancers and can be classified into three main histological subtypes: adenocarcinoma (LUAD) (~40% of NSCLC cases), lung squamous cell carcinoma (LUSC) (~30% of NSCLC cases) and Large-Cell Carcinoma (~10–15% of NSCLC cases). In LUAD, epidermal growth factor receptor (*EGFR*) mutations (~15%) and anaplastic lymphoma kinase (*ALK*) rearrangements (~5%) are common actionable oncogenic alterations. Other targetable alterations include *G12C-KRAS* [2], *MET*, *BRAF/MEK*, *HER-2*, *ROS* and *RET* [3]. In approximately 40-50% of patients with NSCLC, no driver mutations have been identified [3, 4].

Different studies have shown that cancer vulnerabilities can be exploited by targeting signaling networks that sustain cell proliferation [5]. In this regard, epigenetic drugs may sensitize cancer cells to established treatments by a variety of mechanisms, such as facilitating the drug’s access to the DNA, re-expressing silenced tumor suppressor genes, rewiring cancer cell metabolism, increasing reactive oxygen species (ROS), disrupting the anti-/pro-apoptotic signaling, increasing the expression of targetable proteins and potentiating the immune system [6]. In preclinical experiments, the effect of first-generation epigenetic drugs in lung cancer was limited or transient, but combination with chemotherapy or targeted therapy resulted in additive or synergistic effects, probably by a “priming” mechanism on cancer cells that facilitates cytotoxicity [7]. For example, the DNA methyltransferase inhibitor (DNMTi) 5-azacitidine acted synergistically with cytarabine and etoposide in NSCLC cell lines [8] and the histone deacetylase inhibitor (HDACi) romidepsin enhanced the antitumor effect of erlotinib in NSCLC cell lines of different histology and mutation status, including *EGFR*- and *KRAS*-mutants, and wild type cell lines, as well as reduced tumor burden in NCI-H1299 xenograft models [9]. However, the use of DNMTi or HDACi alone or in combination showed somewhat disappointing results in clinical trials [7]. This situation has prompted the development of new epigenetic drugs and biomarkers of response, as well as novel combinatorial approaches that may exploit tumor vulnerabilities [10].

Among the emerging epigenetic targets, the histone methyltransferase G9a, encoded by the Euchromatic Histone-Lysine N-methyltransferase-2 (*EHMT2*), has shown to be a relevant target in NSCLC [11]. This enzyme catalyzes mono-methylation and di-methylation of the lysine 9 of histone H3 (H3K9me1 and H3K9me2) and its overexpression in cancer alters the cell genetic program leading to tumor growth, acquisition of a cancer stem cell (CSC) phenotype, invasion, metastasis and drug resistance [12]. In NSCLC, knock-down of *G9a* using shRNAs reduced malignancy in cell lines, including the CSC phenotype, and inhibited tumor growth in xenograft models. However, the use of the G9a inhibitor UNC0642 showed a modest antitumor growth in vivo [13].

Recently, the novel small molecule CM-272, which co-targets G9a and DNMT1, developed in our Institution, has been tested in hematologic tumors, hepatocellular carcinoma and bladder cancer models [14–16] This double-targeting agent has been highly successful in inhibiting tumor growth in these models both in vitro and in vivo, causing cell proliferation inhibition and triggering cell death. We hypothesized that the double targeting of G9a/DNMT1 in human and murine NSCLC models would have a strong impact on tumor growth in combination with other therapies, as a result of CM-272-mediated gene expression reprogramming leading to chemosensitization.

In this study, we first show that high expression of *G9a* and *DNMT1* is associated with poor prognosis in NSCLC patients. In NSCLC experimental models we have found that G9a/DNMT1 inhibition impairs cancer cell malignancy and reduces tumor growth, an effect that is mediated by enzymes AOX1 and SCARA5. Combination of CM-272 with cisplatin, trametinib or vorinostat produced synergistic anticancer effects in a variety of NSCLC cells. CM-272+cisplatin in vivo reduced synergistically tumor growth without toxicity. In addition, we have found that expression and DNA methylation of *SCARA5* could be used in solid and liquid biopsies as a surrogate indicator of tumor G9a/DNMT1 activity.

## Materials and methods

### Cohort of NSCLC patients and sample collection

In a retrospective study, our cohort included 100 patients with resectable NSCLC from the General University Hospital of Valencia (GUHV-Cohort) collected from July 2004 to July 2013. Detailed clinical and pathological information is shown in Supplementary Table 1. Samples were selected following eligibility criteria: resected non-pretreated stage I to IIIA patients (according to the American Joint Committee on Cancer staging manual) with a histological diagnosis of NSCLC. Peripheral blood samples were collected in 10 mL-EDTA tubes before surgical resection. Tumor samples and non-malignant adjacent tissue were obtained at the time of surgery and preserved in RNAlater (Applied Biosystems, USA) at -80 °C until analysis. This study was conducted in accordance with the Declaration of Helsinki and was approved by the ethical review board of the GUHV. All patients signed an informed consent prior to sample collection. Demographic and clinicopathological characteristics of all patients were collected. Follow-up was performed according to the institutional standard for resected NSCLC.

### Cell lines

Thirty-eight human and eight murine NSCLC cell lines were used in our study (Supplementary Table 2). We selected 3 human (H23, H358 and A549) and 1 murine (Lacun3; previously characterized [17]) cell lines for functional assays. Human cells were obtained from ATCC and authenticated by STR profiling. Cells were grown in either RPMI-1640 (Gibco) or DMEM (Gibco) supplemented with 10% of HyClone Serum (Thermo Scientific), 1% penicillin-streptomycin (Lonza), at 37 °C in 5% CO2 humidified atmosphere.

### Cytotoxicity and combination assays

Drug cytotoxic effects were studied with the MTS (3-(4,5-dimethylthiazol-2-yl)-5-(3-carboxymethoxyphenyl)-2-(4-sulfophenyl)-2H-tetrazolium) method (Thermo Scientific) using 96-well plates (BD Falcon) and 48 h incubation with the drugs at different concentrations. Absorbance was read at 490-650 nm in a SPECTROstar Nano (BMG LABTECH) reader with the MARS Data Analysis Software (BMG LABTECH). Cytotoxicity experiments were designed and analyzed following NCI Developmental Therapeutics Program Methodology guides for the NCI-60 Screening [18]. More details can be found in Supplementary Materials and Methods.

### Calculation of cell growth inhibition and drug combination index

To assess the synergistic potential of the drugs, the freely available software CompuSyn was used [19]. This program allows the calculation of the combination index (CI)-isobologram equation and provides a quantitative determination of drug interactions, where CI < 1, =1, and >1 indicate synergism, additive effect, and antagonism, respectively, based on the Chou-Talalay methodology. Dose-response matrices for visualizing the percentage of affected cells per each drug combination were performed with the R package SynergyFinder [20]. 3D-surface plots representing the CI at different drug concentrations were done using plotly R package (https://plotly-r.com).

### Crystal violet and apoptosis assays

For crystal violet assays, 15,000 A549, H358, H23 and Lacun3 cells per well were seeded in 48-well plates (BD Falcon), allowed to attach overnight and treated with CM-272 in combination with different concentrations of either cisplatin, trametinib or vorinostat for 48 h. After this time, cells were fixed with 4% formaldehyde (Panreac) and stained with 1% crystal violet (Sigma-Aldrich).

Apoptosis analysis was carried out by flow cytometry after treating cells with CM-272 for 48 h, using the double staining Annexin-V and SYTOX^TM^ Green, following previously published protocols [21]. Samples were analyzed in a FACS Canto II cytometer (BD Bioscience) and data were processed with the FlowJo software.

### Immunofluorescence

H23 cells were seeded in 4 chamber slides (BD) and treated with CM-272 for 24–48 h. Cells were fixed with 4% methanol-free formaldehyde for 15 min and then rinsed with TBS-0.05% Tween20. Unspecific binding and permeabilization, performed for 30 min RT with 2% BSA-0.3% Triton X-100-PBS and incubation with primary antibody (J2 #76651) at 1:100 dilution in 2% BSA-PBS, was performed overnight at 4 °C. After washing with TBS-0.05% Tween20, Alexa fluor 488 secondary antibody at 1:200 dilution was used for 2 h at RT. Slides were then washed with TBS-0.05% Tween20 and incubated with Spectral DAPI (Akoya) for 5 min. ProLong Diamonf antifade mounting media (Thermo Fisher) was used to coverslip slides and images were taken at 40x in a confocal microscope LSM 800 with Airyscan (Zeiss).

### DNA methylation and digital droplet PCR (ddPCR)

Global methylation was assessed by pirosequencing of the repetitive element *LINE1*. Genomic DNA was extracted using a DNA Kit (Nucleo Spin Tissue, Macherey-Nagel) following the manufacturer’s instructions. DNA purity and concentration were measured using a NanoDrop spectrophotometer (Thermo Scientific). One µg genomic DNA was bisulfite treated and modified using a CpGenome DNA modification Kit (Chemicon International) following the manufacturer’s instructions. After bisulfite modification, “hot start” polymerase chain reaction (PCR) (PyroMark PCR Kit, Qiagen) was performed with denaturalization at 95 °C for 15 min, followed by 45 cycles consisting of denaturation at 94 °C for 1 min, annealing at 55 °C for 1 min, and extension at 72 °C for 1 min followed by a final 10-min extension. PCR primers were as follows: Forward: 5′-TTTTGAGTTAGGTGTGGGATATA-3’; reverse: 5′-Biotin- AAAATCAAAAAATTCCCTTTC-3′. The resulting biotinylated PCR products were immobilized to Streptavidin Sepharose® High Performance beads (GE Healthcare) and processed to yield high-quality ssDNA using the PyroMark Vacuum Prep Workstation (Biotage) according to the manufacturer’s instructions. The pyrosequencing reactions were performed using the PyromarkTM ID (Biotage), and sequence analysis was performed using the PyroQ-CpG analysis software (Biotage).

DNA from tumor samples was obtained with the TriReagent (Sigma, USA) kit. Isolation of circulating-free DNA (cfDNA) from 1 ml of plasma was performed with QIAamp Circulating Nucleic Acid Kit. DNA was quantified with the Qubit system and bisulfite conversion using the EZ DNA Methylation-Lightning kit (Zymo Research, USA) following manufacturer’s recommendations. Methylation analysis was carried out by digital droplet PCR (ddPCR) using the QX200 System (Bio-Rad, Hercules, CA, USA). The list of primers for ddPCR can be found in Supplementary Table 3. ddPCR was evaluated in 19 patient’s samples, in which both tumor and liquid biopsies were available. For demethylation experiments, cells were treated with 5-Aza-2′-deoxycytidine (Sigma) at 5 μM for 72 h in quadruplicates and pellets were collected for RNA and DNA extraction. More details can be found in Supplementary Materials and Methods.

### Chromatin Immunoprecipitation

Cells were exposed to CM-272 for 48 h or left untreated (control) and chromatin-protein cross-linking was carried out by adding 1% of formaldehyde to 10^7^ cells in suspension, followed by incubation for 15 minutes at RT. Cross-linking was quenched by adding 125 mM glycine. The cell pellet was washed twice with PBS, resuspended in SDS lysis buffer and sonicated for 30 s pulses at 30 s intervals. Nanodrop was used to quantify the DNA, in order to perform the immunoprecipitation with the same amount of chromatin. H3K9me2 (ab1220, Abcam) antibody was added to 400 ng of sonicated DNA, incubated overnight at 4 °C and chromatin was isolated by addition of Protein A agarose beads (16-157, Sigma Aldrich). After isolation, DNA was washed and extracted with phenol-chloroform. *AOX1* and *SCARA5* DNA sequences were then amplified by qPCR and data are represented as 2^-((DCt ChIP-Input) - (DCt ChIP Treatment- ChIP Control))^. The sequences of the primers were as follows: *AOX1* forward: GATGCCAATTGCACTCTTCA; reverse: TTTCACTTGCCATACCTTAGAACA; *SCARA5* forward: TCACCAACAAACAGGAATGC; reverse: TCAGGGGCATCAGAGAAATC. qPCR samples were loaded into a 2.3% agarose gel to visualize specific bands and controls. To avoid band saturation and ensure proper visualization, we performed a shorter qPCR run with 35 cycles. A total of 25 µL of PCR product were loaded and electrophoresed for 1 h at 110 V.

### Transcriptomic analysis, quantitative PCR and Western blotting

The transcriptomic profiles of H358 and A549 cells treated with CM-272 were compared to that of untreated cells. Treatment was performed with the CM-272 half-maximal inhibitory concentration (IC_50_) for 48 h and total RNA was extracted with the NucleoSpin® RNA kit (Macherey-Nagel), quantified by Qubit (ThermoFisher) and analyzed for purity and integrity with Experion (BioRad). RNA-seq was carried out at the Genomic Unit of the Spanish National Center for Cardiovascular Research (CNIC, Madrid, Spain). Two hundred ng of total RNA were used to generate barcoded RNA-seq libraries using the NEBNext Ultra RNA Library preparation kit (New England Biolabs). More details on the protocol are described in Supplementary Material and Methods. Raw data on the RNAseq performed in our study can be accessed through the NCBI-GEO (GSE273400) public repository.

qPCR and Western blot from cell extracts were performed following standard protocols. Details can be found in Supplementary Materials and Methods. Primer sequences of the genes studied are shown in Supplementary Table 4. qPCR for *SCARA5* was performed in 94 available tumor samples. For Western blotting, the primary antibodies are specified in Supplementary Table 5.

### Silencing and overexpression of *SCARA5* and *AOX1* genes

To knock-down *SCARA5* and *AOX1* expression, shRNA targeting these genes were constructed by cloning the target sequences into the pLKO.1-puro vector (Addgene: 8453). As control vector we used the empty pLKO.1-puro plasmid. The RNAi Consortium Collection tool (Broad Institute, Public TRC Portal) was used to select four different shRNA sequences per gene (Supplementary Table 6). To produce lentiviruses, HEK293T cells were transfected with the shRNA-containing plasmids, using MISSION Lentiviral packaging mix (Sigma) and X-Tremegene HP DNA transfection reagent (Roche diagnostics). Cells were selected with puromycin (5 μg/mL, Sigma-Aldrich).

To overexpress *AOX1* and *SCARA5*, cDNA sequences were cloned into the doxycycline-inducible lentiviral vector pcw57.1 (addgene:#41393). The cloning process was outsourced to GenScript. The final vectors, pcw57.1-SCARA5 and pcw57.1-AOX1, were verified for correct sequence and orientation through Sanger sequencing. H358 and A549 cells were infected with pcw57.1-SCARA5 or pcw57.1-AOX1 lentiviral particles in complete media, with polybrene added to enhance infection efficiency. After 72 hours, infected cells were selected using puromycin. *SCARA5* or *AOX1* overexpressing clones were established after 2 weeks of puromycin selection. Increased levels of *SCARA5* and *AOX1* were confirmed by qPCR after 48 hours of doxycycline treatment.

### In vivo experiments

The therapeutic efficacy of CM-272 was first tested in subcutaneous models using A549 (5 × 10^6^ cells/ mouse in 6-week-old Rag2^−/−^ female mice) and Lacun3 (1 × 10^6^ cells/mouse in immunocompetent 6-week-old BALB/C female mice). Once tumors reached ~50 mm^3^, animals were randomized in two groups (*n* = 7 per group): treated intraperitoneally with either 5 mg/Kg of CM-272 (5 days a week) or PBS (controls, Ctrl; 5 days a week). The investigator was blinded to the group allocation until treatment was initiated. For micro-positron emission tomography (micro-PET) with the radiotracer 18-fluorodeoxyglucose (18-FDG), mice were fasted overnight but allowed to drink water *ad libitum*. Uptake was evaluated by maximum standardized uptake value (SUVmax), metabolic tumor volume (MTV) and total lesion glycolysis (TLG) parameters. These experiments were carried out at the micro-PET Core Facility, Nuclear Medicine Department, University Clinic of Navarra.

To test the efficacy of CM-272 in an orthotopic and immunocompetent context, 5 × 10^5^ Lacun3-GFP-LUC expressing cells were injected in the tail vein of 6-week-old BALB/C female mice to allow cell engraftment in the lungs (*n* = 7 animals per group). Tumor burden was monitored with the PhotonIMAGER^TM^ OPTIMA system for quantification of luciferase activity, which was measured after injecting 1.5 mg of D-luciferine (Sigma) diluted in 100 μL PBS intraperitoneally, at the time of cell inoculation (Time 0) and once a week. Analysis of tumor load was performed with the M3 Vision software.

Finally, to test the combination of CM-272 and cisplatin, 5×10^6^ A549 cells were inoculated subcutaneously and once tumors reached ~50 mm^3^ in volume, 6-week old female animals were randomized in four different groups: Control (Ctrl; PBS-injected, IP five days a week), CM-272 (5 mg/Kg, IP five days a week), cisplatin (3 mg/Kg, IP every 3 days) and combination (CM-272+cisplatin, with the same treatment schedule as for individual treatments) (*n* = 7 mice per group). Animal procedures were carried out according to the Institutional Animal Care and Use Committee (IAUC) of the University of Navarra under a protocol approved by the regional Government of Navarra.

### Bioinformatic and statistical analyses

Data normalization and processing for the RNA-seq transcriptomic analyses were carried out at CIMA Bioinformatics Platform using R and Bioconductor open softwares. Background correction and normalization were performed with RMA (Robust Multichip Average) algorithm and counts were normalized using edgeR and voom, as previously described [21]. The functional enrichment of the gene rankings was analyzed with Gene Set Enrichment Analysis (GSEA) [22]. RNASeq2 level III RSEM data from the international initiative “The Cancer Genome Atlas” (TCGA), was obtained using the TCGA2STAT R package [23].

In gene expression analyses, comparisons between non-malignant and malignant samples were assessed with the Mann-Whitney U test. The Kaplan–Meier plotter tool (https://kmplot.com) and Log-rank test were used to study the prognostic value of G9a (*EHMT2*), *DNMT1*, *AOX1* and *SCARA5*, using the median as cut-off. Pearson’s or Spearman’s tests were used for correlation analyses.

For promoter methylation analysis, TCGA data as well as data from the previously published CURELUNG cohort [24] of NSCLC tumors (*n* = 444 patients) and non-malignant samples (*n* = 25) were used. A subset of this cohort, comprising 198 surgically resected NSCLC patients, was also used to assess the prognostic value of *SCARA5* and *AOX5* DNA promoter methylation. To classify patients according to their methylation levels, a β-value threshold of 0.5 was considered, and the Mann–Whitney *U* test or calculation of the cumulative differences in the percentage of methylation for each consecutive CpG was used for comparisons. Correlation between methylation and expression levels was tested with Pearson’s or Spearman’s tests. Survival analysis was assessed with Kaplan-Meier and Log-rank tests. Prognostic value of methylation status was analyzed with Cox regression models. Relapse-free survival (RFS) was estimated as the time from surgery to recurrence, and overall survival (OS) as the time from diagnosis to the date of death or the patient’s last follow-up.

Data for differential expression of *AOX1* and *SCARA5* in normal vs. tumor samples and correlation analyses between the expression of *G9a*, *DNMT1*, *AOX1* and *SCARA5* were done using publicly available datasets, accessed through the “lung cancer explorer” web portal (https://lce.biohpc.swmed.edu/lungcancer/) [25]. Expression of *AOX1* and *SCARA5* upon treatment with decitabine was assessed using RNAseq data from GSE145663 [26].

Other statistical analysis, mainly related to in vitro and in vivo experiments can be found in Supplementary Materials and Methods.

## Results

### Expression and prognostic significance of *DNMT1* and *G9a* in NSCLC

**Fig. 1:**
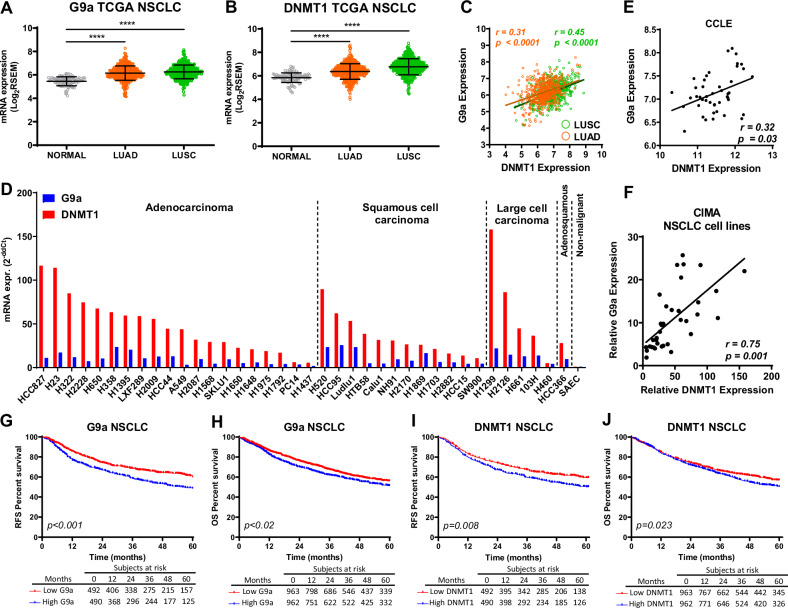
G9a and DNMT1 levels in NSCLC. **A**, **B** Comparison of G9a (A) and DNMT1 (B) mRNA expression between non-malignant lung tissue and LUAD or LUSC patients (TCGA). U-Mann Whitney was used for the statistical analysis. **C** Correlation analysis between G9a and DNMT1 expression in NSCLC patients from TCGA. G9a and DNMT1 levels are highly correlated in LUAD and LUSC. **D** DNMT1 and G9a mRNA levels evaluated by qPCR, in a panel of 38 NSCLC cell lines from our institution (CIMA). **E**, **F** Correlation analysis between G9a and DNMT1 expression in 45 lung cancer cell lines from the Cancer Cell Line Encyclopedia (CCLE)(E) and in our collection of cell lines (evaluated by qPCR)(F). **G**, **H** Kaplan–Meier curves show that NSCLC patients with higher G9a (above the median) levels are associated with worse relapse-free survival (RFS)(G) and overall survival (OS)(H). **I**-**J** Similarly, higher DNMT1 levels (above the median) are associated with lower RFS (I) and OS (J). Comparisons between non-malignant and malignant samples were assessed with the Mann–Whitney *U* test; Pearson’s and Spearman’s correlation (**E**, **F**), and Kaplan–Meier and Log-rank tests (**G**–**J**) were also used. ****p < 0.0001.

We first assessed whether *G9a* and *DNMT1* expression was different in tumor specimens from NSCLC patients and in non-malignant lung samples. Both LUAD and LUSC showed significantly higher (*p* < 0.0001) levels of *G9a* and *DNMT1* than non-malignant lung tissues (Fig. 1A-B; TCGA cohort). As these enzymes play a coordinated role, we also analyzed if there was a correlation between the expression of both genes. Using patient’s TCGA data we found a highly significant positive correlation between *G9a* and *DNMT1* for both LUAD and LUSC (Fig. 1C). Quantification of *DNMT1* and *G9a* mRNA levels in a panel of 38 human NSCLC cell lines by qPCR revealed, as a general trend, higher levels of *DNMT1* than *G9a*. Expression of both genes was higher in cancer cells than in non-malignant cells (SAEC cells) (Fig. 1D). A significant positive correlation between *G9a* and *DNMT1* expression was also found in NSCLC cell lines, when the evaluation was carried out with data from the cancer cell line encyclopedia (CCLE) database or when qPCR was used in the cells available in our laboratory (Fig. 1E-F). Finally, using publicly available data from the Kaplan-Meier plotter, we evaluated the prognostic potential of *DNMT1* and *G9a* expression in NSCLC. Establishing the median mRNA expression as cut-off, we found that patients with high *DNMT1* or *G9a* levels were significantly associated with shorter RFS and OS than those with low levels (Fig. 1G-J). Collectively, these data support a co-targeting approach of DNMT1 and G9a in NSCLC.

### Dual targeting of DNMT1 and G9a impairs proliferation and triggers apoptosis in NSCLC cell lines

**Fig. 2:**
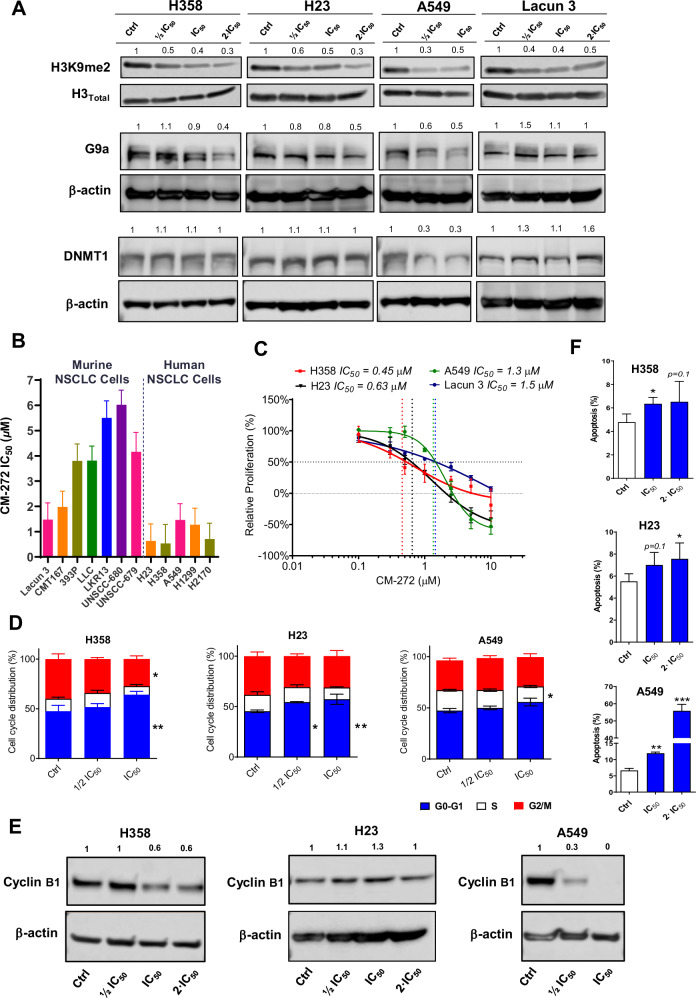
Effect of CM-272, a G9a/DNMT1 inhibitor, on NSCLC cells. **A** Representative blots showing that CM-272 decreases levels of the G9a downstream target H3K9me2 in H358, H23, A549 and Lacun3 cells. G9a levels are also reduced in the human cell lines and DNMT1 levels remain in general constant, except for A549 cells, where a reduction with doses ½ IC_50_ and IC_50_ is observed. The dose corresponding to 2·IC_50_ is not shown for A549 cells due to massive cell death, which precluded obtaining enough proteins. **B**, **C** Cytotoxicity screening in a panel of human and murine cell lines to test CM-272 sensitivity. CM-272 IC_50_ (**B**) shows that CM-272 has a higher cytotoxicity in human cell lines. Curves for H358, A549, H23 and Lacun3 using different doses of CM-272 (C) show a dose-dependent cytotoxic effect elicited by CM-272. **D** Effect of CM-272 on phases of the cell cycle for H358, H23 and A549 cells compared to untreated control cells. **E** Representative western blots for cyclin B1 upon treatment with different doses of CM-272. **F** Evaluation of apoptosis in H358, H23 and A549 cells exposed to CM-272. Western blots were performed at least twice. Numbers above the bands reflect quantification of each band after normalization with β-actin levels in the representative blot. Data for controls (Ctrl) were set at 1 and intensities corresponding to the different CM-272 concentrations are referred to those of the control. Comparisons between groups were performed with one-way ANOVA. *p < 0.05; **p < 0.01; ***p < 0.001.

We next evaluated protein expression of H3 lysine 9 di-methylation (H3K9me2) in four different adenocarcinoma cell lines (H358, H23, A549 and Lacun3) as a readout of the inhibitory enzymatic activity of CM-272. Levels of G9a and DNMT1 were also studied. Doses of ½ IC_50_, IC_50_ and 2·IC_50_ of CM-272 were tested, except for A549 cells, where the massive cell death elicited by 2·IC_50_ doses precluded its analysis at this concentration due to lack of enough protein. Reduced levels of H3K9me2 were observed for all cells upon CM-272 administration, following a dose-response pattern (Fig. 2A). G9a levels were dose-dependently reduced in the 3 human cell lines, but not in Lacun3 cells. DNMT1 levels remained in general constant, except for A549 cells, where a reduction with ½ IC_50_ and IC_50_ doses was observed (Fig. 2A). LINE1 pyrosequencing was also performed in H358 cells after IC_50_ CM-272 administration for 10 days (with drug replacement every 48 h), as a readout of global methylation changes. Pyrosequencing analysis was focused on 5 CpGs of *LINE1* and the treatment with CM-272 led to partial hypomethylation (∼7–12%) of these CpGs (Supplementary Fig. 1A). To study in vitro effects of G9a/DNMT1 co-targeting on cell proliferation we selected a panel of five NSCLC human cell lines (H23, H358, H2170, A549 and H1299) and seven murine NSCLC cell lines (Lacun3, CMT167, 393 P, LLC, UNSCC-679, UNSCC-678 and LKR13). CM-272 decreased cell proliferation in both subsets of cell lines, but lower IC_50_ values were found for the human cells (Fig. 2B). This could be related to the higher affinity of CM-272 for the human enzymes [14]. Representative dose-response curves for H358, A549, H23 and Lacun3 cells are shown in Fig. 2C.

To characterize the effect of CM-272 on cell growth dynamics in vitro, we performed cell cycle analysis in H358, H23 and A549 cells (Fig. 2D). These experiments revealed a significantly higher percentage of cells in the sub-G_0_/G_1_, 48 h upon CM-272 administration, for H358 (Ctrl vs. IC_50_; *p* < 0.01) and H23 (Ctrl vs. ½ IC_50_; *p* < 0.05 and Ctrl vs. IC_50_; *p* < 0.01) cells. Modest changes in the cell cycle were observed for A549 cells, but a statistically significant reduction of cells in the S-phase was found (Ctrl vs. IC_50_; *p* < 0.05). A lower proportion of H358 cells in the G2/M phase was also obtained when the IC_50_ dose was used (*p* < 0.05) (Fig. 2D). Western blot analyses of cyclin B1 showed reduced levels in H358 and A549 cells, but not in H23 cells (Fig. 2E).

Flow cytometry assays with Annexin-V and Sytox green staining to quantify apoptosis in H358, H23 and A549 cells showed in general an increase in apoptotic rates upon addition of CM-272 for 48 h (Fig. 2F). Significant differences were observed for H358 (Ctrl vs. IC_50_; *p* < 0.05), H23 (Ctrl vs. 2·IC_50_; *p* < 0.05) and A549 cells (Ctrl vs. IC_50_; *p* < 0.01, Ctrl vs. 2·IC_50_; *p* < 0.001). A tendency towards significance was also found for some of the doses used in H358 (Ctrl vs. 2·IC_50_; *p* = 0.1) and H23 (Ctrl vs. IC_50_; *p* = 0.1). Taken together, these results show that CM-272 treatment hampers cell proliferation and induces programmed cell death in NSCLC cells. In H358 and H23 cells, CM-272 seems to play mainly a cytostatic effect, whereas in A549 cells, a cytotoxic effect leading to considerable apoptosis is observed. An example of flow cytometry plot showing Annexin-V and Sytox green staining can be found in Supplementary Fig. 1B.

### CM-272 decreases in vivo tumor growth in human and murine NSCLC models

**Fig. 3:**
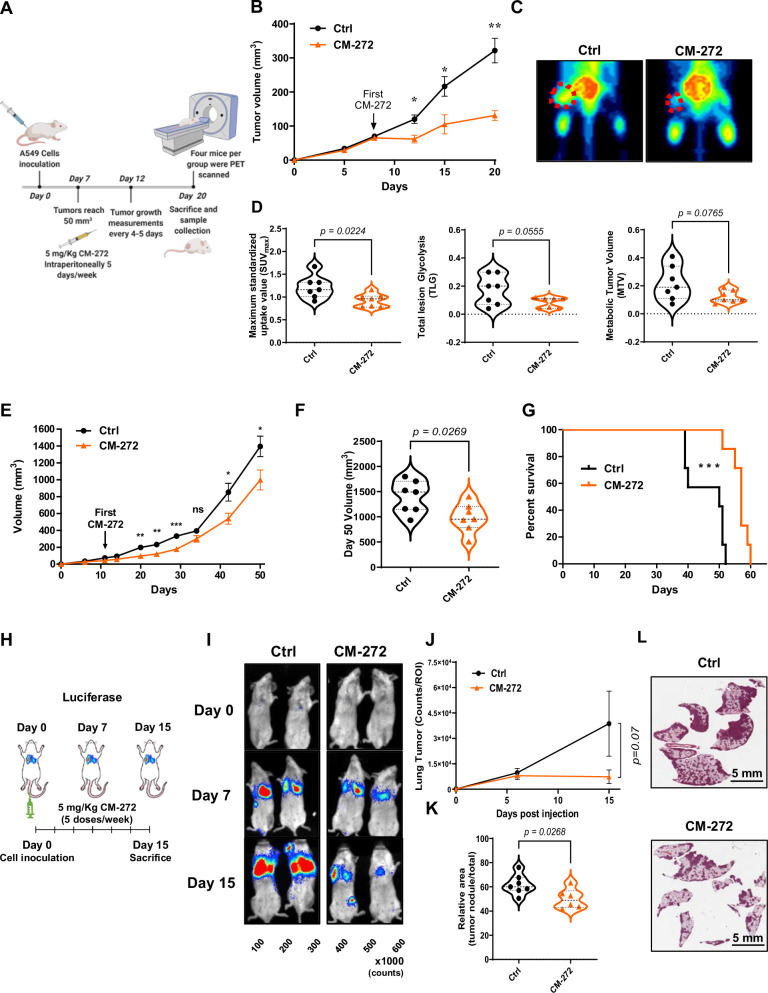
Effect of CM-272 in vivo, in NSCLC models. **A** Experimental design used for the treatment of A549-bearing mice (subcutaneous injection) with CM-272 (5 mg/Kg 5 days a week) and evaluation of response (*n* = 7 mice per group). **B** Tumor volume was reduced by 55% (*p* < 0.001) in treated mice, compared with controls. **C** Representative images of micro-PET analysis from control and CM-272-treated animals. **D** Quantification of maximum standardized uptake values (SUVmax), total lesion glycolysis (TLG) and metabolic tumor volume (MTV) from micro-PET data. **E** Tumor volume in Lacun3-bearing mice (Balb/C background; subcutaneous injection) administered with CM-272 (5 mg/Kg 5 days a week) and untreated controls (*n* = 7 mice per group). **F** Dot plot representing tumor volume for each individual mouse at the end of the experiment (day 50 post-injection). An average of 30% reduction (*p* < 0.05) in tumor growth was found in this model, in comparison with controls. **G** Survival curves showed a significant increase (*p* < 0.001) in survival in mice treated with CM-272, with respect to controls. **H** Scheme showing the experimental design to test the effect of CM-272 in an orthotopic context, using tail vein injection and luciferase expressing-Lacun3 cells (*n* = 7 mice per group). **I** Representative images of luciferase activity at days 0, 7 and 15. **J** Quantification of thoracic luminometric signals measured as counts/ROI throughout the experiment. **K** Quantification of the malignant area vs. total lung area by histological image analysis. **L** Representative H&E images of lungs from control and CM-272-treated animals. Ctrl: untreated controls. Comparisons between the study groups were performed with Student”s *t* test. For the survival analysis (**G**), the Log-rank test was used. *p < 0.05; **p < 0.001; ***p < 0.001.

Further evaluation of the antitumor potential of G9a and DNMT1 inhibition was carried out using A549 human and Lacun3 murine models. In the first one, aimed to assess whether dual inhibition of G9a and DNMT1 affected tumor growth and metabolic uptake, we used immunocompromised mice, where A549 cells were subcutaneously injected (Fig. 3A–D). Cells were inoculated at day 0, and tumor growth was followed up until an average volume of 50 mm^3^ was reached. At that point, CM-272 was administered intraperitoneally 5 days per week, at the dose of 5 mg/kg (see the experimental setting in Fig. 3A). In agreement with our in vitro results, CM-272 showed antitumor efficacy in vivo, with a ~55% (*p* < 0.001) tumor volume reduction at the end of the experiment (Fig. 3B). To assess tumor metabolic activity, 18-FDG uptake was quantified by microPET (Fig. 3C, D). A significant decrease was found in maximum standardized uptake value (SUVmax, by 23%; *p* = 0.015), and a tendency close to statistical significance was observed for metabolic tumor volume (MTV, CM-272 reduction by 41%; *p* = 0.08) and total lesion glycolysis (TLG, CM-272 reduction by 42%; *p* = 0.056) in mice receiving the drug. In Fig. 3C, representative micro-PET images of control and CM-272 treated mice are shown.

We also tested the effect of CM-272 on the highly aggressive model Lacun3. Using subcutaneously injected cells, we found a significant reduction in tumor volume (*p* < 0.05), although less pronounced than that for the human model (Fig. 3E, F). Survival of treated mice was also significantly increased (*p* < 0.001) (Fig. 3G). In a second experiment, we evaluated the effect of CM-272 after inoculation of Lacun3 GFP-LUC-transduced cells in the tail vein (the experimental design is shown in Fig. 3H). Luciferase activity, as surrogate marker of orthotopically grown tumors, was monitored for two weeks. As shown in Fig. 3I-J, CM-272 decreased the lung luminometric signal, a reduction that was close to statistical significance (*p* = 0.07), when compared to that of controls at day 15. Tumor burden was also quantified by image analysis in histological sections of lungs from these animals. The area occupied by tumors was significantly reduced (*p* < 0.05) in animals treated with CM-272 than in controls (Fig. 3K, L).

### CM-272 synergizes with chemotherapy, targeted therapy and epigenetic therapy in NSCLC

Based on our results showing that CM-272 had an antiproliferative effect on NSCLC cells and altered the cell cycle, and that some epigenetic drugs can act as cancer drug sensitizers [10, 27] we wondered whether G9a/DNMT1 blockade would synergize with other cancer drugs. To test this hypothesis, we used three different approaches: chemotherapy with cisplatin, the standard of care in advanced NSCLC patients; targeted therapy with trametinib, a MEK inhibitor downstream of KRAS; and the epigenetic drug vorinostat, a pan-HDAC inhibitor. We first assessed H358, H23, A549 and Lacun3 cells sensitivities to cisplatin, trametinib and vorinostat alone, in order to calculate the IC_50_ (Supplementary Fig. 1C). From this screening we concluded that Lacun3 cells were sensitive to cisplatin and trametinib, and mostly resistant to vorinostat. In the case of the three human cell lines, all of them were sensitive to cisplatin and vorinostat, but were highly resistant to trametinib, without reaching the IC_50_, except for H23 cells (Supplementary Fig. 1C).

**Fig. 4:**
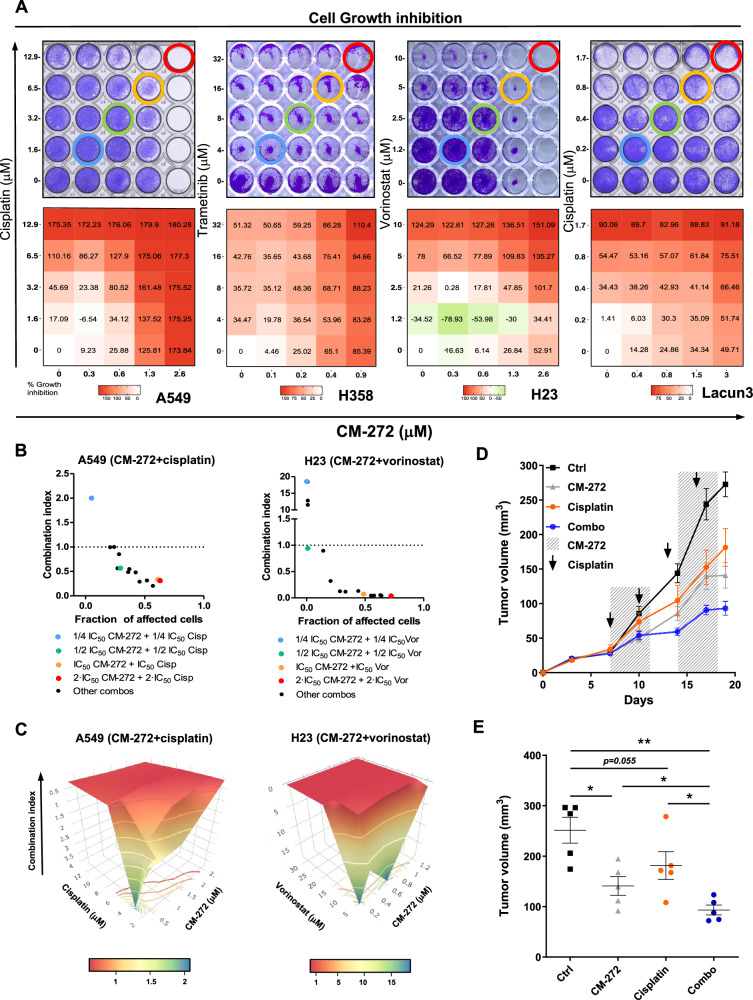
Drug combinations between CM-272 and cisplatin, trametinib or vorinostat. **A** Representative images of crystal violet staining and dose-response matrices to assess the cytotoxic effects caused by combination between CM-272 and either cisplatin (A549 and Lacun3), trametinib (H358) or vorinostat (H23). Doses correspond to ¼ IC_50_, ½ IC_50_, IC_50_ and 2·IC_50_ of each drug and CM-272. In dose-response matrices, a more intense red color indicates increased cytotoxicity. Values above 100% correspond to concentrations able to kill the cells. **B** Representation of combination index (CI) vs. fraction of affected cells (fa) in some of the combination experiments using A549 and H23 cells. The colored dots represent the combination between the indicated doses of each drug and CM-272. Black dots represent all the other possible dose combinations. **C** Examples of 3D-surface plots for combination experiments to better visualize synergies. The *X*, *Y* and *Z* axes represent the concentration of CM-272, the concentration of the other drug used and the previously calculated CI, respectively. The orange-red color indicates higher synergy score, measured by the combination index. **D** Tumor growth curves of the in vivo experiment to evaluate the antitumor effect of the combination CM-272+cisplatin, or the single drugs in the A549 subcutaneous model, compared to untreated controls. **E** Dot plot representing tumor volume for each mouse at day 19. A significant reduction in tumor volume (*p* < 0.05) was found when comparing single treatments with the combination schedule. *N* = 7 animals per group. Comparisons between groups were performed with one way ANOVA. *p < 0.05, **p < 0.01.

We then designed the combination experiments: doses of ¼ IC_50_, ½ IC_50_, IC_50_ and 2·IC_50_ of either cisplatin, trametinib or vorinostat were used in combination with ¼ IC_50_, ½ IC_50_, IC_50_ and 2·IC_50_ of CM-272. Assays were conducted by MTS and crystal violet staining and data were analyzed using different bioinformatic tools. We found that all these combinations had synergistic/additive effects in all the cells tested (Fig. 4 and Supplementary Figs. 2–4). Representative crystal violet assays and dose-response combination matrices performed with the R package SynergyFinder, show in Fig. 4A synergistic effects of CM-272+cisplatin in A549 and Lacun3 cells, CM-272+trametinib in H358 cells and CM-272+vorinostat in H23 cells. Other examples of synergistic combinations are shown in Supplementary Fig. 2–4). Calculation of the combination index (CI) using CompuSyn, and 3D-surface plots assessed with plotly R package confirmed the synergism of the combinations. Representative examples are depicted in Fig. 4B, C for the combination CM-272+cisplatin in A549 cells and CM-272+vorinostat in H23 cells.

Based on the in vitro results we decided to validate the therapeutic potential of combining CM-272 with cisplatin in vivo using the A549 subcutaneous model. Mice received either vehicle, CM-272, cisplatin or the combination CM-272+cisplatin. As shown in Fig. 4D, E, both CM-272 and cisplatin in monotherapy reduced tumor growth by 35–40% at the end of the study. The CM-272+cisplatin combination achieved an average of ~65% decrease in tumor growth. In Fig. 4E, dot plot of tumor volumes corresponding to the last day of the experiment revealed that differences between single treatments and the combined treatment were statistically significant (*p* < 0.05 for both drugs). Of note, no toxicities were found for any treatment (as evaluated by mice weight and histopathological examination of liver and kidneys; not shown).

### CM-272 elicits major transcriptomic changes in A549 and H358 cells

**Fig. 5:**
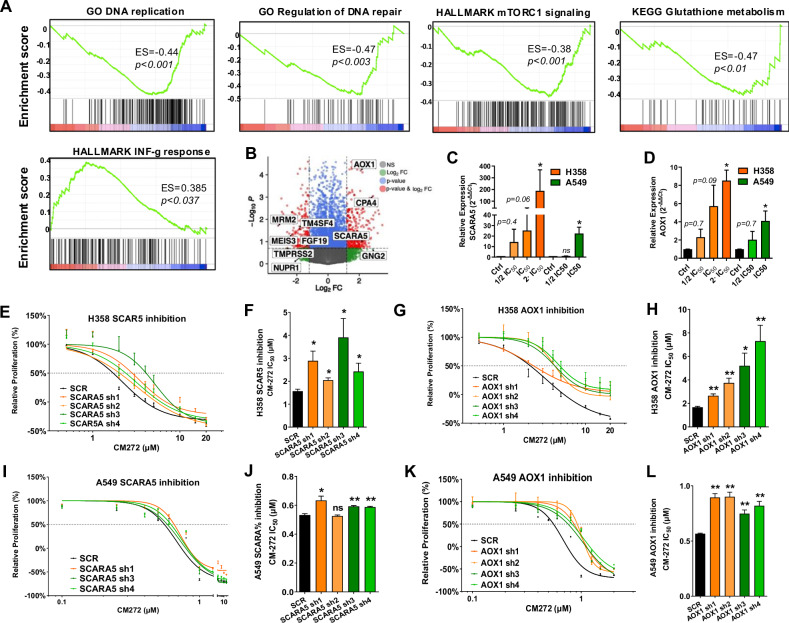
Transcriptomic changes elicited by CM-272 in NSCLC cells. **A** GSEA analyses showing significant negative enrichment of “DNA replication”, “Regulation of DNA repair”, “mTORC1 signaling” and “Glutathione metabolism”, as well as significant positive enrichment in “INF-γ response”, in both H358 and A549 cells treated with CM-272. **B** Volcano plot showing average Log_2_FC expression changes of deregulated genes in both cell lines treated with CM-272, compared to untreated controls. Some functionally relevant genes are highlighted. This analysis allowed the identification of *AOX1* and *SCARA5* as upregulated genes with a possible role in the cytotoxic activity of CM-272 in treated cells. **C**, **D** Dose-dependent increase of *SCARA5* (**C**) and *AOX1* (**D**) expression in H358- or A549-CM-272-treated cells was validated by qPCR. **E** Dose response curves of the cytotoxic effect elicited by CM-272 in H358 control cells (Scr: transduced with the scramble sequence) and in the clones where *SCARA5* was inhibited by each of the 4 shRNAs. **F** IC_50_ values for each of the curves shown in E. Inhibition of *SCARA5* significantly increases the IC_50_ of CM-272 in H358-treated cells. **G**, **H** Dose–response curves (**G**) and IC_50_ values (**H**) for H358 clones when *AOX1* was inhibited by shRNAs. Similar to *SCARA5*, inhibition of *AOX1* in H358 cells increases significantly the IC_50_ of CM-272. **I**, **J** Dose–response curves (**G**) and IC_50_ values (**H**) for *SCARA5* inhibition in A549 cells, compared to controls. The IC_50_ of CM-272 was also higher in the case of shRNA clones, although the effect was milder than that observed for H358. **K**, **L** Dose response curves (**K**) and IC_50_ values (**L**) for *AOX1* inhibition in A549 cells, compared to controls. Similar to H358 cells, CM-272-mediated cytotoxicity in A549 cells was significantly lower in *AOX1*-inhibited clones than in controls. Comparisons between groups were performed with one-way ANOVA. *p < 0.05, **p < 0.01.

RNAseq analysis was performed to deepen into the intrinsic molecular mechanisms altered by CM-272. A549 and H358 cells were used for the transcriptomic analysis because of their high sensitivity to CM-272 in terms of growth inhibition (H358) or apoptosis (A549). Treatment of cells with CM-272 markedly affected transcriptomic profiles in both cell lines: 877 upregulated and 402 downregulated genes were observed for H358, and 702 upregulated and 1021 downregulated genes for A549 (Log_2_FC ± 1.5). Considering only those genes that were consistently up or downregulated in both cell lines we found 110 genes that fulfilled this condition (Supplementary Fig. 5A). Gene set enrichment analysis (GSEA) identified the following significant gene set enrichments in CM-272-treated cells: negative enrichment in GO categories “DNA replication” and “regulation of DNA repair”; negative enrichment in Hallmark “mTORC1 signaling” and KEGG “glutathione metabolism”; and positive enrichment in Hallmark “INF-γ response” (Fig. 5A). Positive enrichment of GO “Basement membrane” and negative enrichment of GO “Genetic imprinting” and “IL-10 production” were also noted (Supplementary Fig. 5B–D).

Volcano plot analysis (Fig. 5B) and quantitative PCR (for validation of the results, Supplementary Fig. 5E) showed downregulation of tumor-promoting genes in CM-272-treated cells, such as rRNA methyltransferase (*MRM2* also known as *FTSJ2*), involved in DNA replication and repair, suggested to be an oncogene in NSCLC [28]; *TMPRSS2*, a gene associated with tumor malignancy [29]; *CXCL8*, a multifunctional cytokine that modulates tumor proliferation, invasion and migration in an autocrine or paracrine manner, whose expression is induced by *TMPRSS2* [30]; and *FGF19*, a metastatic and cancer-stem cell promoting gene in NSCLC [31, 32]. On the contrary, CM-272 increased levels of genes related to tumor growth suppression: *CPA4* (Carboxypeptidase A4), a negative regulator of the AKT/c-MYC pathway in NSCLC [33] and *GNG2* (G-protein gamma subunit 2), which impairs AKT phosphorylation and cell survival [34] (Supplementary Fig. 5F). We also found upregulation of *SCARA5* (Scavenger Receptor Class A Member 5) and *AOX1* (Aldehyde oxidase 1) (Fig. 5C-D). Both *SCARA5* and *AOX1* are hypermethylated and downregulated in breast, prostate and thyroid tumors, in association with poor prognosis and cancer progression [35–37] *SCARA5* overexpression has been related to reduced cancer malignancy and sensitization to DNA damage-based chemotherapy [38–40] *AOX1* also plays a tumor suppressor role and has been found downregulated in prostate, bladder, ovarian and hepatocellular carcinomas [41, 42] Inhibition of *AOX1* has also been linked to a more effective antitumor therapy in hepatocellular carcinoma by blockade of the PI3K-AKT pathway [43]. Although we noticed that several genes deregulated by CM-272 were related to AKT, no decrease in pAKT levels were detected upon CM272 exposure (Supplementary Fig. 5G).

Among the deregulated transcripts, we identified potential transcription factors involved in CM-272 reprograming (Supplementary Fig. 6A) and increased expression of endogenous retroviral elements (ERVs) and interferon-I-related genes (Supplementary Fig. 6B). This latter finding is in agreement with previous reports using epigenetic drugs in cancer cells, including CM-272 [44]. In line with this result, cytoplasmic dsRNA was observed as a consequence of CM-272 treatment (Supplementary Fig. 6C). ERVs expression has been coupled with necroptotic cell death [44]. We verified increased levels of pMLKL, a hallmark of necroptosis, in cells exposed to CM-272 (Supplementary Fig. 6D). A scheme depicting deregulated genes and biological processes induced by the G9a/DNMT1 inhibitor CM-272 found in this study, is shown in Supplementary Fig. 6E.

### AOX and SCARA5 facilitate response of cancer cells to CM-272

Because it was reported that *SCARA5* and *AOX1* had a negative impact on cancer cell malignancy and tumor growth, improved the efficacy of anticancer drugs and were epigenetically regulated in cancer, we decided to concentrate on these genes, to evaluate whether CM-272-induced expression would participate in the cytotoxic effect of this drug. To address this issue, shRNAs were used to block *SCARA5* and *AOX1* expression and MTS assays were undertaken (Fig. 5E–L). In H358 cells, statistically significant lower cytotoxicity, reflected by higher IC_50_ values, was observed when *SCARA5* or *AOX1* were inhibited with any of the four different shRNAs used. Similar results were obtained when *AOX1* was inhibited in A549 cells, whereas a more modest effect was found for *SCARA5*, with only two shRNAs (sh 1 and sh 4) showing significantly higher IC_50_ values than controls. These data show the involvement of SCARA5 and AOX1 in CM-272-mediated cytotoxic effects. To assess whether these genes directly affected cell growth, we carried out overexpression experiments and clonogenic assays in A549 and H358 cells. No significant changes in colony number or area were observed when *AOX1* or *SCARA5* were overexpressed (Supplementary Fig. 7A-B). This result could suggest that the facilitation of CM-272 cytotoxicity elicited by the expression of these genes is related to mechanisms other than cell proliferation.

### SCARA5 and AOX1 as potential biomarkers in NSCLC patients

**Fig. 6:**
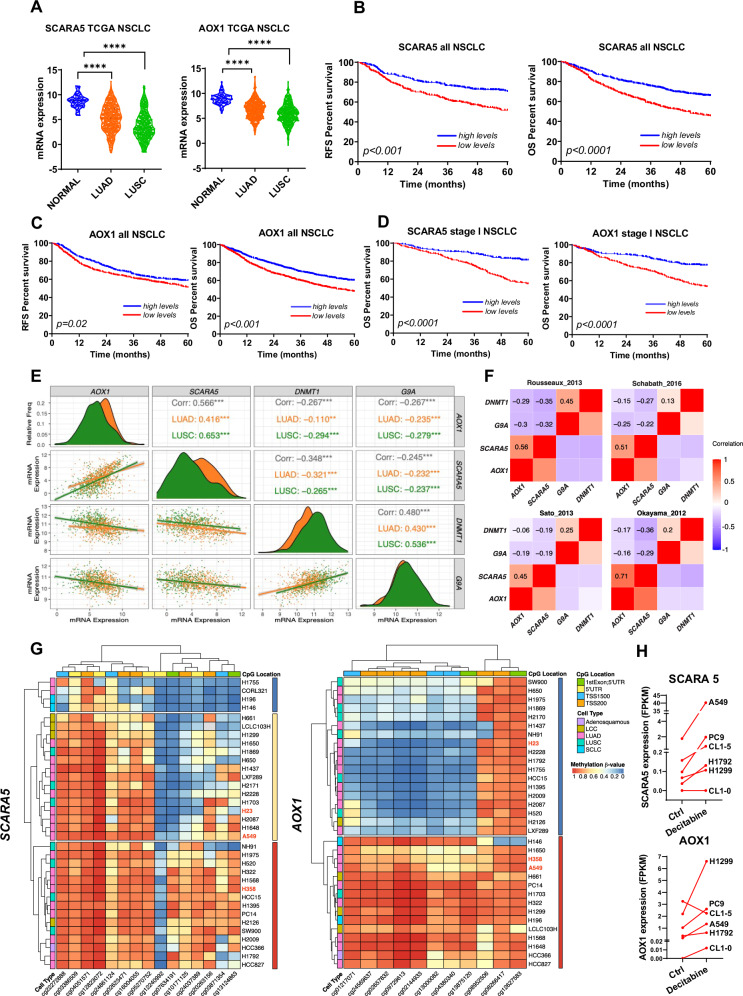
Levels of the CM-272 targets SCARA5 and AOX1 in NSCLC. **A** Both *SCARA5* and *AOX1* mRNA levels are significantly reduced (*p* < 0.0001) in LUAD and LUSC specimens from the TCGA cohort. **B** Kaplan–Meier curves of all stages NSCLC patients to study relapse-free survival (RFS) and overall survival (OS) based on *SCARA5* expression levels. Low levels (red color) of *SCARA5*, considering the median as cut-off, associate with reduced RFS and OS. **C** Similar to *SCARA5*, mRNA levels of *AOX1* below the median (red color) associate with lower RFS and OS in all NSCLC patients. **D** Considering only early stages (stage I), both markers keep their prognostic value for OS. **E** Correlation matrix to evaluate correlation between *AOX1*, *SCARA5*, *DNMT1* and *G9a* expression in NSCLC patients from the TCGA cohort. In the diagonal of the matrix, histograms representing expression densities are shown. At the left/bottom part of the matrix, point distribution and regression curves for either LUAD or LUSC are shown. On the right upper part of the chart, R correlation coefficients resulting from comparisons between gene pairs are shown in black. Correlation coefficients for LUAD and LUSC are also shown in orange and green, respectively. **F** Correlation matrix analyses using publicly available datasets from GEO (Rousseaux-2013; Okayama-2012; Sato-2013; Schabath-2016) to confirm positive correlations between *AOX1-SCARA5* and *DNMT1-G9a* expression, and negative correlations between *SCARA5-G9a*, *SCARA5-DNMT1*, *AOX1-G9a* and *AOX1-DNMT1* expression, in NSCLC patients. **G** Heatmap of CpG b-values for *SCARA5* and *AOX1* promoter methylation in 34 lung cancer cell lines from the CURELUNG dataset, analyzed with the Infinium 450 K array (Illumina). Hierarchical clustering revealed three main methylation clusters for *SCARA5* and two for *AOX1* (see vertical colored bars on the right side of each cluster). The blue color in the cluster indicates hypomethylation and the red color hypermethylation. In the case of *SCARA5*, cluster 1 (blue, 4 cell lines; 11%) was characterized by low DNA promoter methylation levels (<50% of CpGs); cluster 2 (yellow, 15 cell lines; 44%) by intermediate methylation levels (50% to 75% of CpGs); and cluster 3 (red, 15 cell lines; 44%), by high methylation levels (>75% of CpGs). For *AOX1*, cluster 1 (blue, 18 cell lines; 53%) was characterized by hypomethylation of most CpG but consistent hypermethylation of CpGs cg08952506, cg08266417 and cg12627583; and cluster 2 (red, 16 cell lines; 47%) by hypermethylation of virtually all CpGs. **H** In the GSE145663 public dataset, treatment of NSCLC cell lines with decitabine caused up-regulation of both *SCARA5* and *AOX1*. Comparisons between groups were performed with one-way ANOVA. Survival analyses were carried out with Log-rank test and correlation analyses with Pearson/Spearman tests. ****p < 0.0001.

We also hypothesized that SCARA5 and AOX1 would be potential biomarkers in NSCLC patients that could be used in future studies to assess the effect of G9a/DNMT1-targeting drugs. First, we interrogated TCGA data and found that levels of both *SCARA5* and *AOX1* were significantly reduced (*p* < 0.001) in NSCLC tumors compared to non-malignant lung samples (Fig. 6A). This result was also found in 8 different NSCLC datasets and was consistent for both LUAD and LUSC (Supplementary Fig. 7C, D). Using the Kaplan–Meier plotter and establishing the median as cut-off, we found that NSCLC patients with low *SCARA5* and *AOX1* mRNA levels had a significantly shorter RFS and OS than those with high levels (Fig. 6B, C). When only early stages (Stage I) were considered, similar findings were observed for RFS (Supplementary Fig. 8A) and OS (Fig. 6D) in the case of *SCARA5*. The same analysis in *AOX1* revealed significant differences for OS (Fig. 6D) and a non-significant trend for RFS (Supplementary Fig. 8B).

We then evaluated correlation matrices using TCGA data to study the association between the expression of the CM-272 molecular targets (*G9a//DNMT1*), and *SCARA5/AOX1* genes in NSCLC patients (Fig. 6E). A very significant inverse correlation (*p* < 0.001) was observed between expression of *DNMT1-SCARA5*, *DNMT1-AOX1*, *G9a-SCARA5* and *G9a-AOX1*, for both subgroups of NSCLC patients (LUAD and LUSC). We also found that expression of both *AOX1* and *SCARA5* was very positively correlated (*p* < 0.001). Moreover, in 10 different datasets from NSCLC patients, bioinformatic analyses identified a positive correlation between *AOX1-SCARA5* expression, which was inversely correlated with that of *G9a-DNMT1* (Fig. 6F and Supplementary Fig. 8C).

As previously mentioned, expression of both *SCARA5* and *AOX1* has been shown to be regulated by DNA promoter methylation. In spite of the fact that these genes do not harbor a CpG island in the promoter, CpG-enriched areas within the TSS200 transcription start sites, susceptible to DNA methylation, are found. To estimate DNA hypermethylation in our cancer context, we first studied whether treatment with the demethylating agent 5’-azacitidine (5-Aza) increased the expression of *SCARA5* and/or *AOX1*. Supplementary Fig. 9A, B shows significantly increased levels of *SCARA5* (in 7/10 cell lines) and *AOX1* (in 4/10 cell lines) upon treatment with 5-Aza. The up-regulation of *SCARA5* was more pronounced than that for *AOX1*, with >10-fold increase in 6 of the cell lines. We then decided to evaluate the degree of DNA methylation in *SCARA5* promoter, upon administration of 5-Aza or CM-272 (Supplementary Fig. 9C). To this aim, we used methylation-specific ddPCR targeting cg20263156, a CpG we found to be heavily methylated in tumor samples from NSCLC patients. DNA promoter methylation was significantly decreased when cells were treated with 5-Aza, but did not change when exposed to CM-272 (Supplementary Fig. 9C).

Levels of H3K9me2 associated with *SCARA5* and *AOX1* promoters were next evaluated by ChiP analyses in CM-272-treated and untreated H358 cells. As shown in Supplementary Fig. 9D, E, H3K9me2 levels were significantly reduced (p < 0.001) when treated with the G9a/DNMT1 inhibitor.

Using the CURELUNG dataset, we studied *SCARA5* and *AOX1* DNA promoter methylation patterns in a panel of 34 NSCLC cell lines (Fig. 6G). For *SCARA5*, heatmap and cluster analyses showed 15 cell lines (44%) with intermediate (50% to 75% of CpGs; designated as cluster 2) and 15 with high (>75% of CpGs; cluster 3) methylation levels. For *AOX1*, 18 cell lines (53% of CpGs; cluster 1) showed hypomethylation of most CpG but consistent methylation of CpGs cg08952506, cg08266417 and cg12627583; the other 16 cell lines (47%; cluster 2) were characterized by methylation of virtually all CpGs. In the GSE145663 public dataset, treatment of a 6-NSCLC cell line panel with the demethylating agent decitabine caused up-regulation of both *SCARA5* and *AOX1* in all cell lines but one (Fig. 6H).

**Fig. 7:**
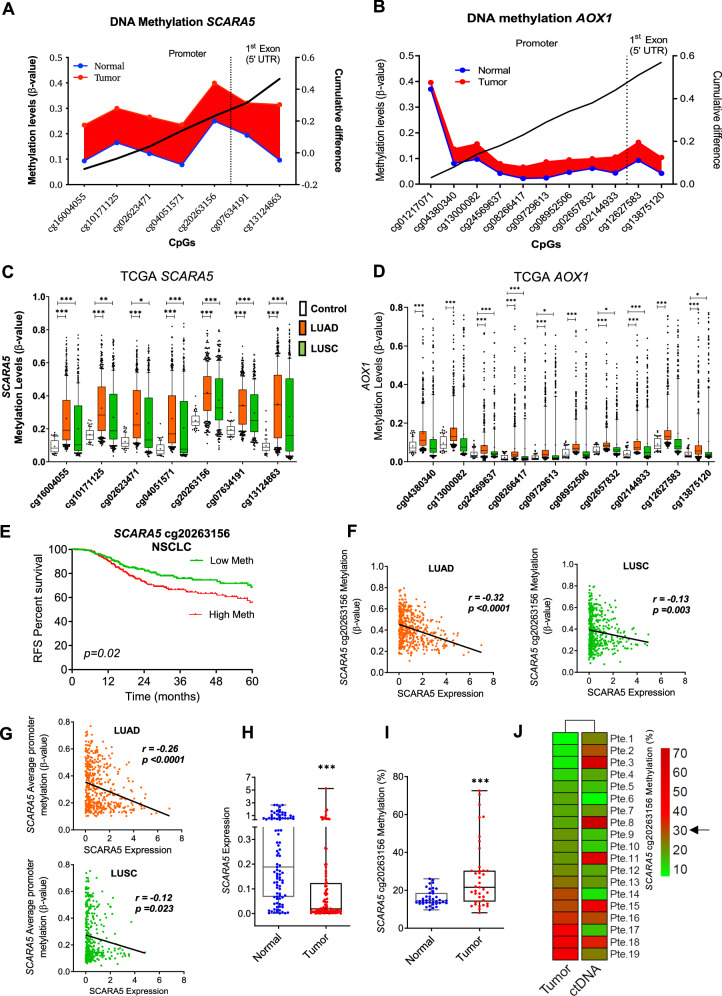
DNA methylation status of SCARA5 and AOX1 in NSCLC. **A**, **B** DNA methylation pattern of the different CpGs found in *SCARA5* (A) or *AOX1* (B) promoters (TCGA dataset). Methylation status in non-malignant samples are shown with a blue line; and in tumor samples, with red line. The red area corresponds to CpGs where there is hypermethylation in tumors, compared to normal samples. **C**, **D** Comparison of methylation status for each of the CpGs from *SCARA5* (**C**) or *AOX1* (**D**) promoters in non-malignant samples, LUAD and LUSC (TCGA). **E** Hypermethylation of *SCARA5* cg20263156 (median as cut-off) associates with significant reduction in relapse-free survival (RFS). **F** *SCARA5* expression and *SCARA5* cg20263156 hypermethylation are inversely correlated in both LUAD and LUSC. **G** Considering the average values of all *SCARA5* CpGs, hypermethylation is also inversely correlated with SCARA5 expression in both histological types. **H** mRNA expression levels found in non-malignant versus malignant samples from the HGUV cohort, quantified by qPCR. **I** *SCARA5* cg20263156 methylation status found in non-malignant versus malignant samples from the GUHV cohort, quantified by methylation-specific ddPCR. **J** Methylation of cg20263156 was studied in both tumors and cfDNA from a sub-cohort of GUHV NSCLC patients, by methylation-specific ddPCR. Color codes represent the percentage of methylation (green: low methylation; red: high methylation) found in tumors and in ctDNA. The arrow represents the methylation levels (30%) above which we consider an abnormal methylation. Concordant results are identified in 14/19 samples (73.68%), and discordant results in 5/19 (26.31%). Comparisons between non-malignant and malignant samples were estimated with the Mann–Whitney *U* test. Survival was assessed with the Log-rank test and correlation analyses with Pearson/Spearman correlation tests. Time-to-event was assessed also with Cox regression analysis. *p < 0.05, **p < 0.01; ***p < 0.001.

To explore the potential of *SCARA5* and *AOX1* as epigenetic biomarkers we evaluated the methylation degree of each promoter CpG in NSCLC patients from the TCGA and CURELUNG NSCLC cohorts. Both *SCARA5* and *AOX1* showed hypermethylation in tumor samples compared to non-malignant lung samples (Fig. 7A, B). Hypermethylation was found for both LUAD and LUSC, and was particularly consistent for *SCARA5*, where all CpGs spanning from cg16004055 to cg13124863 were methylated (Fig. 7A–D). These results were validated in the CURELUNG cohort (Supplementary Fig. 9F, G).

We then studied whether there was an association between hypermethylation of each of the CpGs within *SCARA5* or *AOX1* promoters and RFS in both TCGA and CURELUNG cohorts. OS was not evaluated because data on survival are not available in the CURELUNG dataset. A significant association between hypermethylation and lower RFS was found for *SCARA5* CpG cg20263156, in both cohorts (Fig. 7E and Supplementary Fig. 9H). High DNA methylation of cg02623471 and cg04051571 was also associated with reduced RFS, but only in the CURELUNG dataset (Supplementary Fig. 9H). No prognostic value was found for any of the *AOX1* CpGs (not shown).

Uncropped western blots included in this study can be found in Supplementary Fig. 10, with the exception of H3K9me2, because histone extracts were purified for the blot, which is expected to provide clean bands.

As cg20263156 showed potential as biomarker, we focused on this CpG. The degree of *SCARA5* cg20263156 DNA methylation was inversely correlated (*r* = −0.32; *p* < 0.0001) with *SCARA5* expression in NSCLC tumors, in particular with LUAD (*r* = −0.32; *p* < 0.0001, LUSC; *r* = −0.13; *p* < 0.003) (Fig. 7F). Similar findings were observed when the average methylation of all CpGs within the promoter was considered (Fig. 7G). Significantly reduced mRNA levels of *SCARA5* (*p* < 0.0001) (Fig. 7H) and increased levels of *SCARA5* cg20263156 DNA methylation (*p* < 0.0001) (Fig. 7I) were validated in our GUHV cohort by qPCR and ddPCR, respectively. Considering data from ddPCR, TCGA and CURELUNG, we considered >30% promoter cg20263156 DNA methylation to be abnormal, as such percentages were not found in 95% of non-malignant lung specimens. We also studied whether *SCARA5* DNA methylation could be detected in circulating-free DNA (cfDNA) in a sub-cohort of GUHV where we had both tumor and plasma samples available. Comparing results in tumors with those obtained in ctDNA from their respective patient, concordant results were identified in 14/19 samples (73.68%), and discordant results in 5/19 (26.31%) (Fig. 7J).

## Discussion

Historically, the use of first-generation epigenetic drugs (i.e. azacytidine, decitabine), alone or in combination with chemotherapy, showed limited activity in solid tumors, including NSCLC. However, numerous clinical trials with newer epigenetic agents targeting a variety of enzymes are underway, mainly in combination with other cancer drugs [10]. In the present study we have tested preclinically the therapeutic effect of co-targeting epigenetic enzymes G9a and DNMT1 in NSCLC. We have first shown that expression of both genes is highly increased in LUAD and LUSC patients, in association with poor prognosis. Moreover, expression of G9a and DNMT1 is significantly correlated in both NSCLC patients and cell lines, which provides a rationale for the co-targeting strategy. We have also shown that dual G9a/DNMT1 blockade plays an antitumor and antimetastatic effect in NSCLC models and that the G9a/DNMT1-targteting drug CM-272 acts as a lung cancer drug sensitizer. G9a/DNMT1 inhibition led to reduced proliferation, alteration of cell cycle and induction of apoptosis/necroptosis in NSCLC cell lines.

In our study, a combination of CM-272 with either cisplatin, trametinib or vorinostat resulted in synergistic cytotoxic effects with most of the drug concentrations tested. Moreover, in the A549 in vivo model we have demonstrated that CM-272 combined with cisplatin exerts synergistic anticancer effects. The synergy between CM-272 and cisplatin or immunotherapy has also been found in bladder cancer [15] and hepatoblastoma models [45]. Similarly, CM-272 showed potent antitumor effects when combined with proapoptotic agents (Bcl-2 inhibitor venetoclax, BCL-XL inhibitor A1331852, MCL-1 inhibitor S63845) in multiple tumor types [44]. These data suggest that the transcriptomic reprogramming of cancer cells elicited by G9a/DNMT1 blockade can sensitize tumors to different cancer drugs that act through unrelated molecular mechanisms.

Blockade of G9a/DNMT1 led to alteration of several intracellular pathways in NSCLC cells, many of which affect the expression of membrane-bound proteins and extracellular signaling (such as FGF19/FGFR4, TMPRSS2, CXCL8/CXCR1-2, GNG2 and SCARA5). In a liver fibrosis model, it was described that CM-272 plays an antifibrotic role by reprograming extracellular-dependent cascades, more prominently TGF-β and VEGF signaling [16]. In addition, as we have seen in our study, viral mimicry due to expression of ERV/dsRNA and interferon-related genes upon CM-272 treatment, which in turn constitutes stress signals that force necroptotic cell death, have been reported in several studies [44, 46]

Results from our RNAseq analysis also evidenced the upregulation of *SCARA5* and *AOX1* by CM-272 treatment as well. This called our attention because high expression of these genes, which are epigenetically regulated, act as chemosensitizers [35, 37–40, 42]. Functional studies have shown that SCARA5, a class A scavenger receptor related to iron metabolism, interacts with focal adhesion kinase (FAK) and subsequently inhibits the FAK-Src pathway, thus leading to decreased cell growth and invasion [39]. AOX1, a phase I xenobiotic enzyme with a role in oxidation of drugs and toxicants [47], has also been associated with impairment of tumor growth and sensitization to cancer drugs [35]. *AOX1* knock down leads to increased levels of NADP, as well as higher use of the pentose phosphate pathway (PPP) and nucleotide synthesis, thus enabling promoting cell invasion [35]. In our study, blockade of *SCARA5* or *AOX1* reduced cytotoxicity of CM-272, shown by higher IC_50_ values. Intrigued by the regulation of *SCARA5* and *AOX1* expression by G9a/DNMT1 blockade, we investigated possible correlations between these enzymes in NSCLC patients. The highly significant inverse correlation between *G9a/DNMT1* and *SCARA5/AOX1* gene expression could reflect a co-regulated pattern of expression and functionality in patient’s tumors. We then verified that *SCARA5/AOX1* promoters were hypermethylated in tumors and cancer cell lines and that, upon treatment of the cells with 5-azacitidine, both genes were re-expressed. Although G9a/DNMT1 blockade causes re-expression of silenced genes that may be involved in antitumor response [16], direct DNA demethylation by CM-272 was not the cause of *SCARA5* and *AOX1* re-expression in NSCLC cell lines. This result would be in keeping with the modest decrease in global DNA methylation that we have found in NSCLC cells. However, CM-272 reduced levels of the transcriptional repression mark H3K9me2 bound to the promoter regions of *SCARA5* and *AOX1*, which suggests that, at least in NSCLC cancer cells, this may constitute a main mechanism of activity. This contrasts with results published in leukemia cell line models, where CM-272 reduces levels of both global DNA methylation and H3K9me2 levels [15].

Interestingly, low *SCARA5* expression or DNA hypermethylation, as well as low *AOX1* expression were associated with poor prognosis in NSCLC patients in our study. Moreover, we have found that *SCARA5* DNA promoter methylation can be readily detected by ddPCR in plasma samples from NSCLC patients, with levels matching in general those found in their corresponding tumor counterparts. The relevance of this putative epigenetic biomarker should be investigated in prospective trials dealing with G9a/DNMT1 targeting drugs.

## Conclusion

The present work demonstrates that co-targeting G9a/DNMT1 in NSCLC represents a novel antitumor strategy in NSCLC, with SCARA5 and AOX1 as mediators of the cytotoxic activity and *SCARA5* DNA methylation as potential companion biomarker. In addition, G9a/DNMT1 inhibition sensitizes cancer cells to chemotherapy (cisplatin), targeted therapy (trametinib) and epigenetic therapy (vorinostat).

## Supplementary information

Supplemental Material

Original Data

## Data Availability

Raw data on the RNAseq can be accessed through the NCBI-GEO (GSE273400) public repository. Any other information will be available upon request.
