# Supplementary material for: G9a/DNMT1 co-targeting inhibits non-small cell lung cancer growth and reprograms tumor cells to respond to cancer-drugs through SCARA5 and AOX1
Source: Cell Death Dis. 2024 Nov 2;15(11):787. doi: 10.1038/s41419-024-07156-w (PMC11531574; doi:10.1038/s41419-024-07156-w)
Supplement: Supplementary file 1 — Supplemental Material [file 41419_2024_7156_MOESM1_ESM.pdf]

Supplementary materials and Methods

Cytotoxicity and combination assays

Absorbance data were normalized with that obtained from cells at day 0. The following drugs were used *in vitro* to evaluate cytotoxicity: CM-272 (obtained from the Program in Molecular Therapies, CIMA), cisplatin (Sigma), trametinib (Selleckchem) and vorinostat (Cayman Chemical). Cytotoxicity experiments were designed and analyzed following NCI Developmental Therapeutics Program Methodology guides for the NCI-60 Screening.

DNA methylation and digital droplet PCR (ddPCR)

For the amplification reaction, the following conditions were used: 95°C for 10 min, 94°C for 30s, 52°C for 1 min (40 cycles) and 98 °C for 10 min. Data were analyzed with QuantaSoft analysis (BioRad). Methylated and unmethylated controls were added in each plate and water was also included as non-template control. All reactions were performed in triplicates.

RNA-seq and transcriptomic analysis

200 ng of total RNA were used to generate barcoded RNA-seq libraries using the NEBNext Ultra RNA Library preparation kit (New England Biolabs). Briefly, poly A+ RNA was purified using poly-T oligo-attached to magnetic beads followed by fragmentation and then first and second cDNA strand synthesis. Next, cDNA ends were end-repaired and adenylated. The NEBNext adaptor was ligated, followed by uracil excision from the adaptor and PCR amplification. Finally, the size of the libraries was checked using the Agilent 2100 Bioanalyzer DNA 1000 chip and the DNA concentration was determined with the Qubit® fluorometer (Life Technologies). Libraries were

sequenced on a HiSeq2500 (Illumina) to generate 60 bases single reads. FastQ files for each sample were obtained using the CASAVA v1.8 software (Illumina).

Quantitative PCR

For gene expression analysis from patient's tumor samples, RNA was isolated with TriReagent (Sigma) according to the manufacturer's instructions. Reverse transcription (RT) was performed with 500 ng of total RNA using random hexanucleotides and the High-capacity cDNA Reverse Transcription Kit (Applied Biosystems, USA). SYBR Green I-based real-time quantitative polymerase chain reaction (qPCR) method was used to analyze *SCARA5* mRNA levels. Relative gene expression levels were calculated as the ratio of target gene/reference gene (*GUSB*) expression, by normalizing the data with Human Reference cDNA (Clontech, USA).

Statistical analyses

Normality of the data was assessed with the Shapiro-Wilk test. For *in vitro* and *in vivo* experiments, statistical differences between groups were compared with the Student´s t test (two groups) or ANOVA (when comparing several groups). For non-parametric analyses comparing two groups or several groups, the U-Mann Whitney or the Kruskal-Wallis tests were used, respectively. Sample size calculations were done with the G*Power free software. For animal studies, based on previous data from the Laboratory (21), and the expected tumor sizes (300-1000 mm^3), with a standard deviation of 200; to get an expected reduction in tumor growth by 50% approximately; for a confidence level α=0.05 and power (1-β)=80%, the desired effect magnitude would be f=0.68, with a calculated number of animals of 7 per group. For the cohort of patients, taking into account our previous results on NSCLC biomarkers, with a relative risk of 2.2, 80% potency and 95% confidence, the calculated sample size is 90. Our study has increased

the number to a total of 100 patients. No animals or patients were excluded from the analyses. For statistical comparisons, GraphPad Prism 5 (GraphPad, Inc) or Rstudio softwares were used. Values are expressed as means ± SD or means ± SEM, and statistical significance was defined as $p< 0.05$ (*), $p < 0.01$ (**) and $p < 0.001$ (***).

Supplementary Figure legends

Supplementary Figure 1. (A) *LINE1* pyrosequencing performed in H358 cells after IC_{50} CM-272 administration for 10 days (with drug replacement every 48h), as a readout of global methylation changes. Pyrosequencing plot shows 5 CpGs of *LINE1* and partial hypomethylation (~7-12%) of these CpGs when cells are exposed to CM-272. **(B)** Representative images of CM-272 effects on A549 apoptosis, analyzed by flow cytometry, using annexin-V and Sytox. **(C)** Cytotoxic effect of cisplatin, trametinib and vorinostat in Lacun3, H23, H358 and A549 cells. Dose-response curves and IC_{50} values are shown for each cell line.

Supplementary Figure 2. Cytotoxic effects represented by dose-response curves, obtained after combination between CM-272 and cisplatin, trametinib or vorinostat. Doses in the X-axes correspond to ¼ IC_{50}, ½ IC_{50}, IC_{50} and $2 \cdot IC_{50}$ of either cisplatin, trametinib or vorinostat in combination with different doses of CM-272, represented by curves with different colors (as indicated in the legend at the bottom-left corner). Dots represent the average of three independent experiments.

Supplementary Figure 3. Dose-response matrices to assess the cytotoxic effects caused by combination between CM-272 and cisplatin in H23 **(A)** and H358 **(B)** cells; CM-272 and trametinib in H23 **(C)**, Lacun3 **(D)** and A549 **(E)** cells; and CM-272 and vorinostat in Lacun3 **(F)**, H358 **(G)** and A549 **(H)** cells. Doses correspond to ¼ IC_{50}, ½ IC_{50}, IC_{50} and $2 \cdot IC_{50}$ of each drug and CM-272. Red color indicates increased cytotoxicity. Values above 100% correspond to concentrations able to kill the cells and values below 0% are combinations lacking efficacy.

Supplementary Figure 4. Representation of combination index (CI) vs. fraction of affected cells (fa) and 3D surface plots for H358 **(A)**, H23 **(B)**, A549 **(C)** and Lacun3 **(D)**

using drug combinations. In the 3D-surface plots, X, Y and Z axes represent CM-272 concentration, the other drug concentration, and the previously calculated CI, respectively, to graphically visualize synergies (The orange-red color indicates higher synergy scored measured by the combination index). The scale-bar represents the CI.

Supplementary Figure 5. (A) Venn diagram showing the number of coincident deregulated genes in A549 and H358 cells treated with CM-272, considering $-2<Log_2FC>1$. **(B-D)** GSEA analyses showing significant positive enrichment of GO category “Basement membrane” (B) and negative enrichment in categories “Regulation of IL-10 production” (C) and “Genetic imprinting” (D). **(E-F)** qPCRs for the detection of selected genes of the RNAseq in H358 and A549 cells treated with CM-272. **(G)** Western blots to detect phosphorylated (p) AKT and total AKT in H358, H23 and A549 cells treated with CM-272. Western blots were performed twice. Numbers above the bands show quantification of each pAKT band after normalization with AKT and β-actin levels in the representative blot. Data for controls (Ctrl) were set at 1 and intensities corresponding to the different CM-272 concentrations are referred to those of the control. Comparisons between gene expression in the two study groups were performed with Student T test. *: $p<0.05$; **: $p<0.01$; ***: $p<0.001$.

Supplementary Figure 6. (A-B) Cluster analysis of transcription factors (A) and endogenous retroviral elements (ERVs) and interferon-I-related genes (B) deregulated by CM-272 in H358 and A549 cells. **(C)** Example of immunofluorescence for double stranded RNA (dsRNA) in H23 cells treated or untreated with CM-272. **(D)** Levels of phosphorylated MLKL (a hallmark of necroptosis) in H358 and A549 treated with CM-272. Western blots were performed twice. Numbers above the bands show quantification of each pMLKL band after normalization with β-actin levels in the representative blot. Data for controls (Ctrl) were set at 1 and intensities corresponding to the different CM-

272 concentrations are referred to those of the control. **(E)** Summary of changes elicited by CM-272 in NSCLC cells found in our study.

Supplementary Figure 7. (A-B) Clonogenic assay performed in A549 (A) and H358 (B) control cells (Ctrl) and their respective clones overexpressing either *AOX1* or *SCARA5*. Colony area and number are shown. No statistical changes are detected. **(C-D)** Differential expression of *SCARA5* (C) and *AOX1* (D) in NSCLC datasets (including LUAD and LUSC).

Supplementary Figure 8. (A, B) Kaplan-Meier curves of stage I NSCLC patients to study relapse-free survival (RFS) based on *SCARA5* (A) or *AOX1* (B) expression levels. The Log-rank test was used for comparisons. Low levels (red color) of *SCARA5*, considering the median as cut-off, associate with reduced RFS, whereas a non-significant trend was observed for *AOX1*. **(C)** Correlation matrices using publicly available datasets from GEO (Lee-2008[GSE8894]; Tang-2013[GSE42127]; Der-2014[GSE50081]; expO-2019[GSE2109], Botling-2013[GSE37745]; Fujiwara-2012b[GSE20853]) to study correlations between *AOX1*, *SCARA5*, *DNMT1* and *G9a* expression, in NSCLC patients.

Supplementary Figure 9. (A, B) Treatment of NSCLC cell lines with the demethylating agent 5'-azacitidine (5-Aza) in a panel of 8 NSCLC cell lines. A significant increase in *SCARA5* (in 7/10 cell lines) (A) and *AOX1* (in 4/10 cell lines) (B) is found upon treatment with 5-Aza. Student T test was used for comparisons. **(C)** Quantification of cg20263156 methylation within the *SCARA5* promoter using methylation-specific ddPCR, in H358, H23 or A549 cells, treated with either CM-272 or 5-Aza, compared to untreated cells. A significant reduction in DNA methylation is observed for the three cell lines with respect to controls, upon treatment with 5-Aza. On the contrary, no changes are found when cells are exposed to CM-272. One way ANOVA was used for statistical comparisons. **(D)** ChiP

analysis performed in H358 cells to detect levels of H3K9me2 levels bound to the *SCARA5* and *AOX1* promoters after exposure to CM-272. One way ANOVA was used for statistical comparisons. **(E)** qPCR products from the Chip assay run through a 2.3% agarose gel to visualize specific bands and controls. C-: control without antibody; CM-272-: cells without drug treatment (control sample). Input: Control samples, where 50 µL of sonicated genomic DNA was directly isolated and PCR amplified. **(F-G)** Comparison between methylation levels of the different CpGs within the *SCARA5* (F) and *AOX1* (G) promoters, in non-malignant samples, LUAD and LUSC, in the CURELUNG cohort of NSCLC patients. Hypermethylation of the promoter is found for all GpGs in both LUAD and LUSC, in the case of *SCARA5*. For *AOX1*, lower number of CpGs are found hypermethylated within the promoter. The Mann-Whitney U test was used for comparisons. **(H)** Cox regression analyses of NSCLC relapse-free survival based on methylation levels of cg20263156, cg02623471 and cg04051571 (within the *SCARA5* promoter). Hypermethylation is shown in red color and hypomethylation in green color. *: $p<0.05$; **: $p<0.01$; ***: $p<0.001$.

Supplementary Figure 10. Uncropped blots shown in the Figures of this manuscript.

Supplementary Table 1. Clinicopathological characteristics of patients from the GUHV cohort.

HGUV-Cohort	
**N= 100**	
**Age (years)**	**N (%)**
≤65	50 (50)
>65	50 (50)
**Gender**	
Female	9 (9)
Male	91 (91)
**Smoking Status**	
Current	47 (47)
Former	44 (44)
Never	9 (9)
**Histology**	
ADC	49 (49)
SCC	50 (50)
Other	1 (1)
**Stage (TNM7)**	
I	48 (48)
II	30 (30)
III	22 (22)
IV	0
**Adjuvant Therapy**	
No	62 (62)
yes	38 (38)

Supplementary Table 2. NSCLC cell lines used in this study. ADC: Adenocarcinoma; SCC: Squamous cell carcinoma; LC: Large cell carcinoma; ADSC: Adenosquamous carcinoma.

Human cell lines			
**Cell Line**	**Histological subtype**	**Cell Line**	**Histological subtype**
**HCC827**	ADC	**H1437**	ADC
**H23**	ADC	**H520**	SCC
**H322**	ADC	**HCC95**	SCC
**H2228**	ADC	**Ludlu1**	SCC
**H650**	ADC	**HTB58**	SCC
**H358**	ADC	**Calu1**	SCC
**H1395**	ADC	**NH91**	SCC
**LXF289**	ADC	**H2170**	SCC
**H2009**	ADC	**H1869**	SCC
**HCC44**	ADC	**H1703**	SCC
**A549**	ADC	**H2882**	SCC
**H2087**	ADC	**HCC15**	SCC
**H1568**	ADC	**SW900**	SCC
**SKLU1**	ADC	**H1299**	LC
**H1650**	ADC	**H2126**	LC
**H1648**	ADC	**H661**	LC
**H1975**	ADC	**103H**	LC
**H1792**	ADC	**H460**	LC
**PC9**	ADC	**HCC366**	ADSC

Murine cell lines	
**Cell Line**	**Histological subtype**
**LACUN 3**	ADC
**CMT-167**	ADC
**393P**	ADC
**LCC**	ADC
**LKR13**	ADC
**UNSCC680AJ2**	SCC
**UNSCC679AJ2**	SCC

Supplementary Table 3. Primers used for the ddPCR.

Assay	Primers/probes	Sequences (5´-3´)
**SCARA5 Methylation**	Forward	TATTTTAGATTTTATTGTAGAAG
	Reverse	AACTCCTCTAAATACTACCTAAA
	Probe (FAM)	TTTCGTAATTGTTTTAAGAATTTTATT
**SCARA5 Unmethylation**	Forward	TATTTTAGATTTTATTGTAGAAG
	Reverse	AACTCCTCTAAATACTACCTAAA
	Probe (HEX)	TTTTGTAATTGTTTTAAGAATTTTAT

Supplementary Table 4. List of primers used for gene expression studies using qPCR.

DNMT1	FW: GCACCTCATTTGCCGAATAC
	RV: CTCCACCACAATCTTGCTGA
G9a (EHMT2)	FW: AACATCGATCGCAACATCAC
	RV: AGGCAGTTGGAGCTAGAGCA
SCARA5	FW: GGAACATCTCCCTCGCGAAA
	RV: CTCGGTCACCTTTGAACCCA
AOX1	FW: TCATCTAAGGGTCTGGGAGAGT
	RV: TCCAAGGAACGTAGGATCCAG
MRM2	FW: AATGGCGGGGTACTTGAAGC
	RV: CGACACCGGTAACTCTCCAC
CXCL8	FW: TCTGCAGCTCTGTGTGAAGG
	RV: ACTTCTCCACAACCCTCTGC
TMPRSS2	FW: CTGGGGAGGGGAACCTGG
	RV: GGTGGTGACCCTGAGTTCAA
CPA4	FW: CCTGCAGGCCCTTTTAGACA
	RV: CGGCCGGTTTTCAAACGAAT
FGF19	FW: GCTTTCGAGGAGGAGATCCG
	RV: GGGGCGAAGAGAACATGTCA
GNG2	FW: AGCCGCTGCTACACATACTC
	RV: CAGATCTGGCTTAGCTCCCG
GUSB	FW: GAAAATATGTGGTTGGAGAGCTCATT
	RV: CCGAGTGAAGATCCCCTTTTTA
GAPDH	FW: ACTTTGTCAAGCTCATTTCC
	RV: CACAGGGTACTTTATTGATG

Supplementary Table 5. List of antibodies used in this study.

Primary Antibody	Company [Ref]	Species	Dilution
DNMT1	Cell Signaling (5032S)	Rabbit	1:1000
G9a (EHMT2)	Invitrogen (MA5-14880)	Rabbit	1:1000
H3K9me2	Abcam (ab1220)	Mouse	1:2000
H3 Total	Millipore (07-690)	Rabbit	1:50000
dsRNA	Cell Signaling (#76651)	Mouse	1:100
Cyclin B1	Cell Signaling (#12231)	Mouse	1:500
pMLKL	Abcam (ab196436)	Rabbit	1:1000
pAKT (Ser 473)	Cell Signaling (#9271)	Rabbit	1:1000
AKT	Cell Signaling (#9272)	Rabbit	1:1000
βACTIN	Sigma (A2228]	Mouse	1:10000
**Secondary Antibody [Ref]**	**Company**	**Species**	**Dilution**
Anti-IgG of rabbit-HRP[Na934v]	GE Healthcare	Sheep	1:2000
Anti-IgG of mouse-HRP[Na934v]	GE Healthcare	Donkey	1:2000

Supplementary Table 6. shRNAs used to inhibit SCARA5 and AOX1 expression.

Gene	TRC number (GPP Web Portal)
SCARA5 shRNA 1	TRCN0000153674
SCARA5 shRNA 2	TRCN0000157433
SCARA5 shRNA 3	TRCN0000158172
SCARA5 shRNA 4	TRCN0000156727
AOX1 shRNA 1	TRCN0000046045
AOX1 shRNA 2	TRCN0000046047
AOX1 shRNA 3	TRCN0000046043
AOX1 shRNA 4	TRCN0000046044

Supplementary Figure 1 *Exposito et al.*

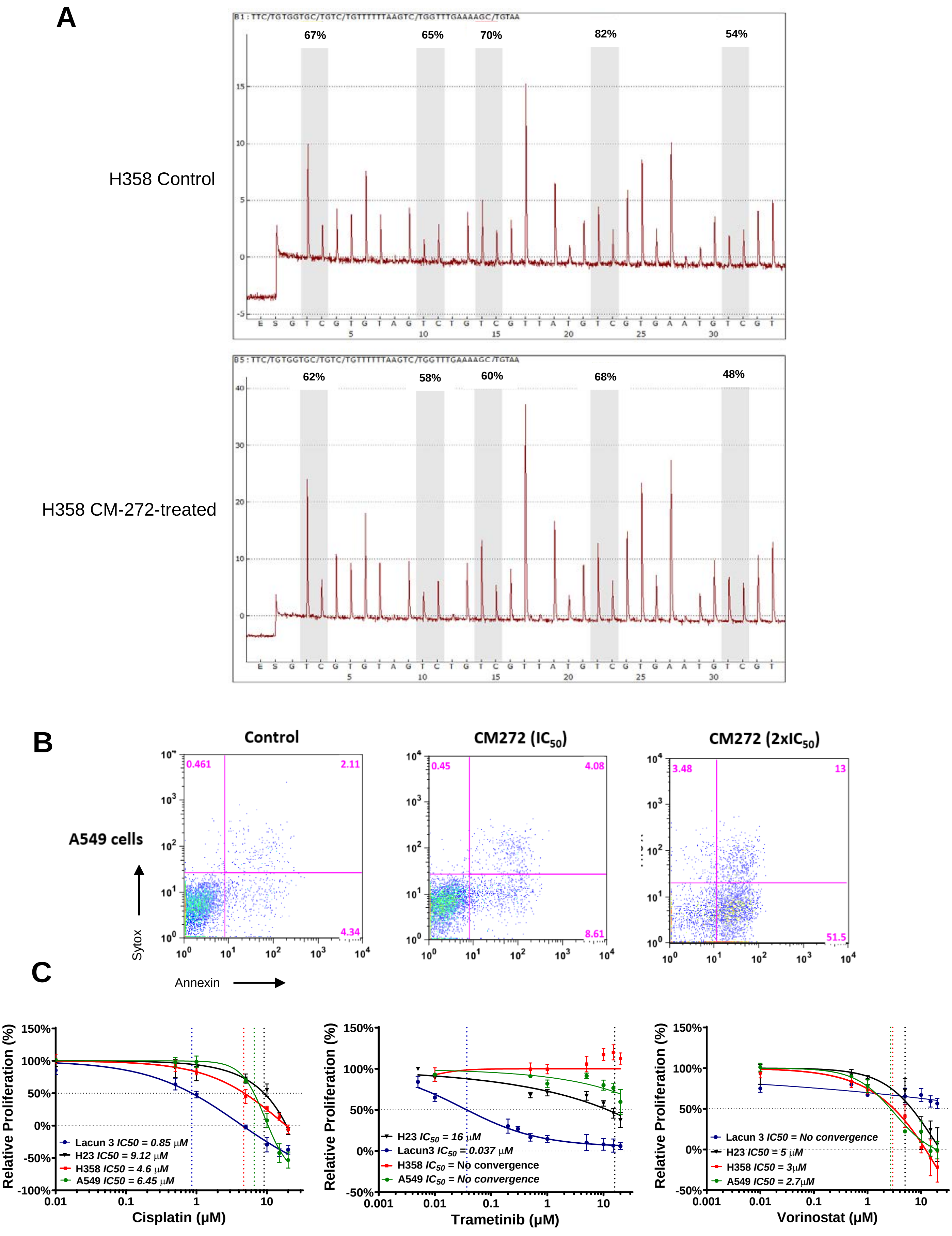

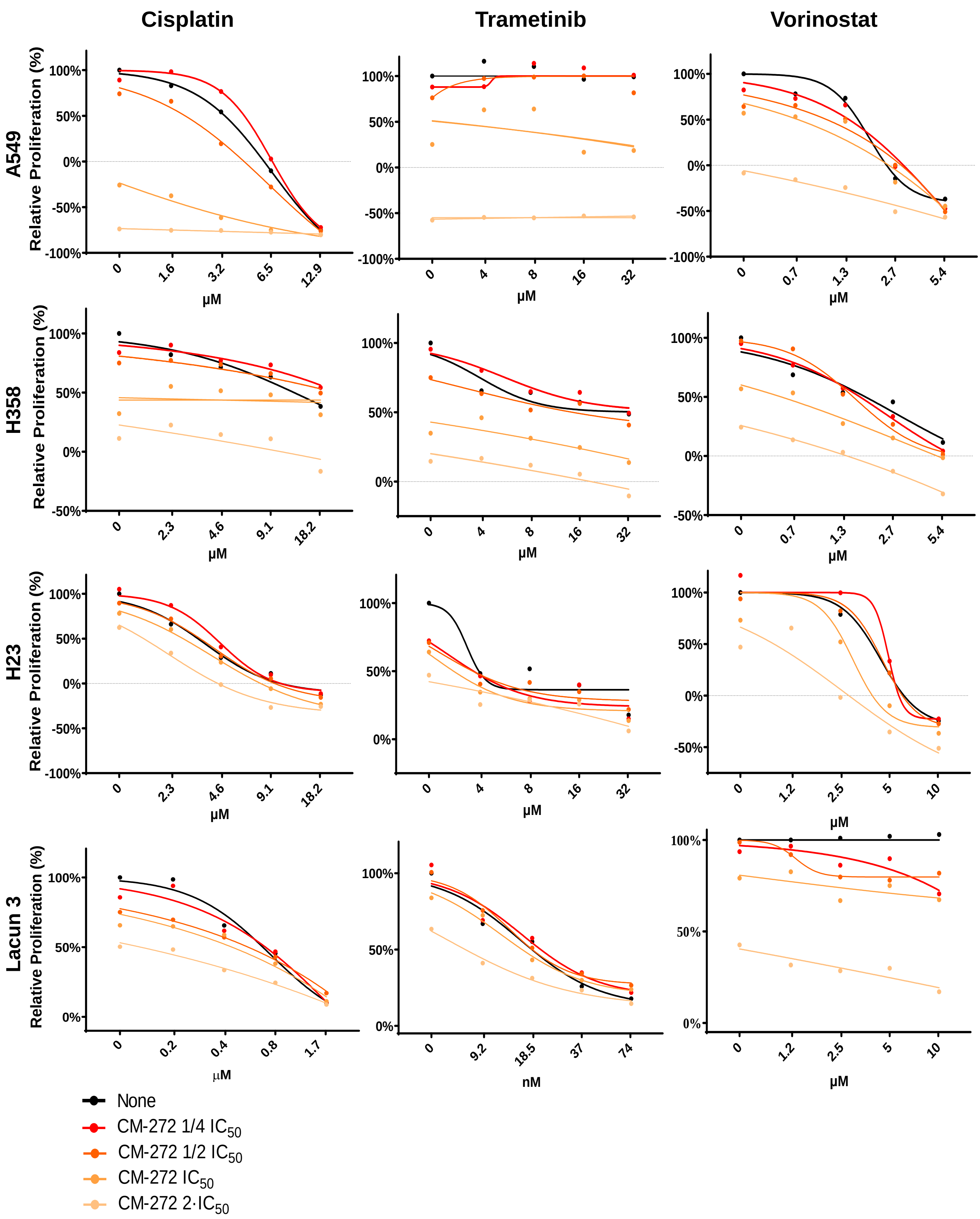
Cisplatin
Trametinib
Vorinostat
A549
H358
H23
Lacun 3
Relative Proliferation (%)
100%
50%
0%
-50%
-100%
0
1.6
3.2
6.5
12.9
4
8
16
32
0.7
1.3
2.7
5.4
2.3
4.6
9.1
18.2
1.2
2.5
5
10
0.2
0.4
0.8
1.7
9.2
18.5
37
74
µM
nM
None
CM-272 1/4 IC50
CM-272 1/2 IC50
CM-272 IC50
CM-272 2·IC50

Supplementary Figure 3 *Exposito et al.*

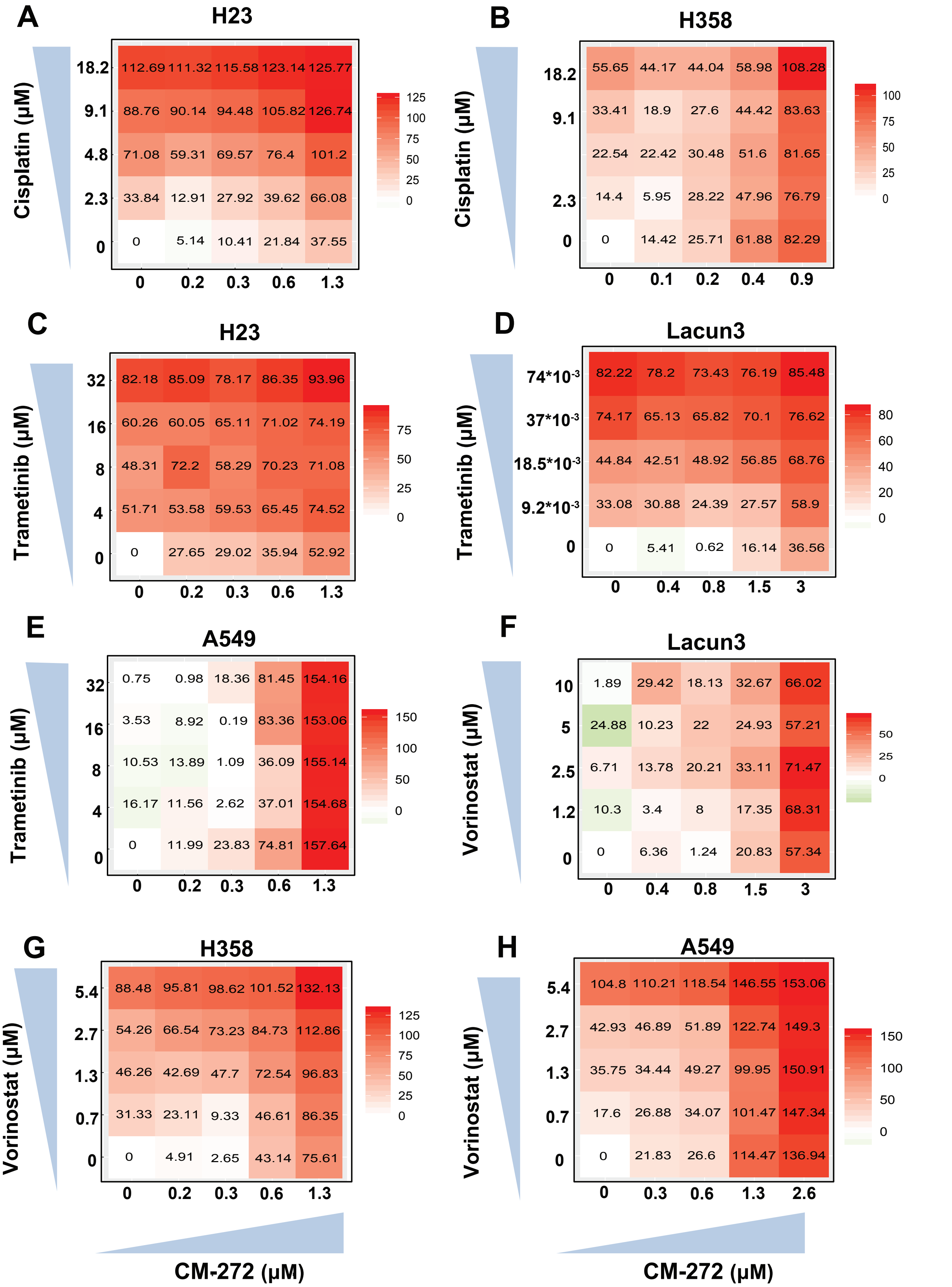

Supplementary Figure 4 *Exposito et al.*

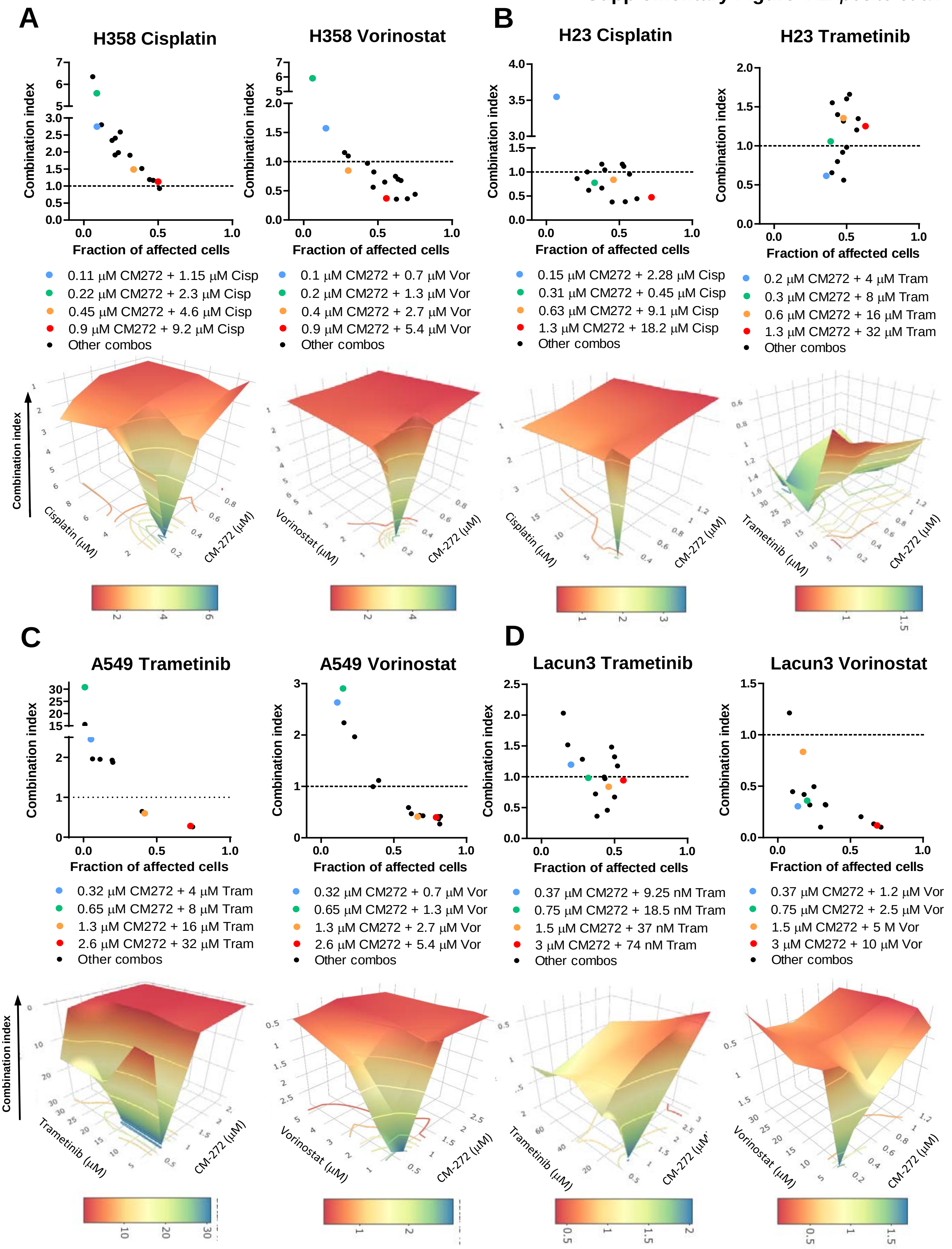

Supplementary Figure 5 *Exposito et al.*

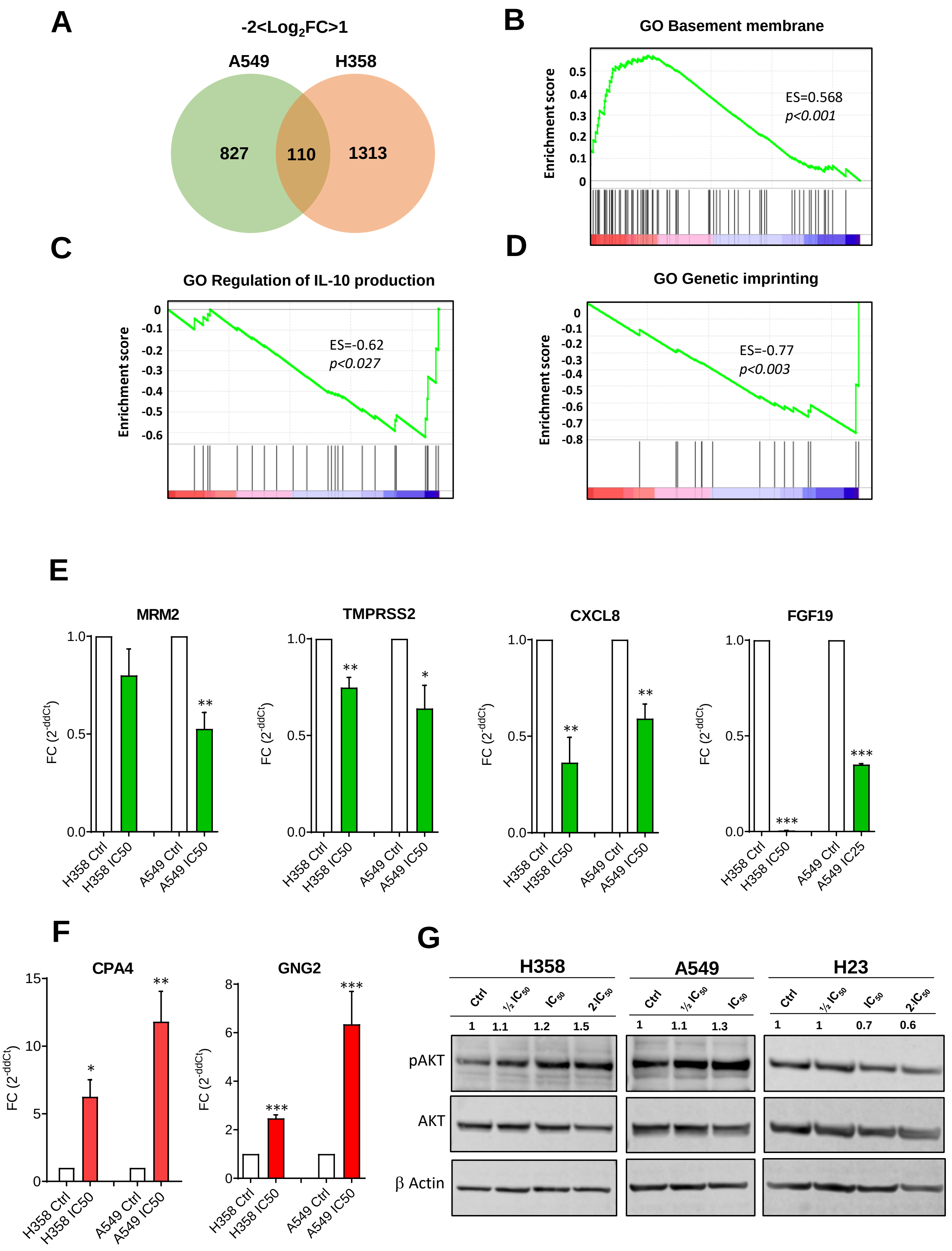

Supplementary Figure 6 *Exposito et al.*

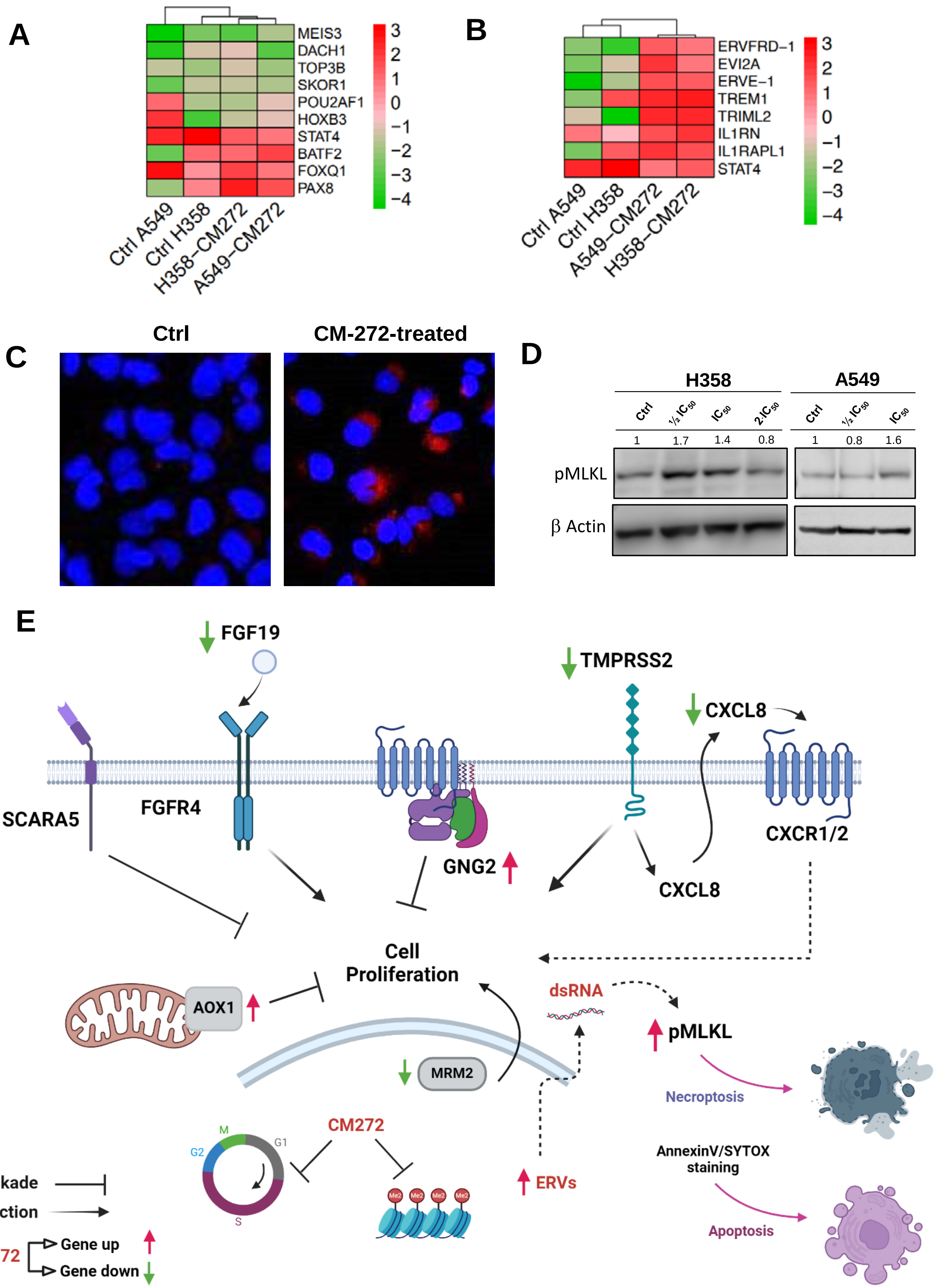

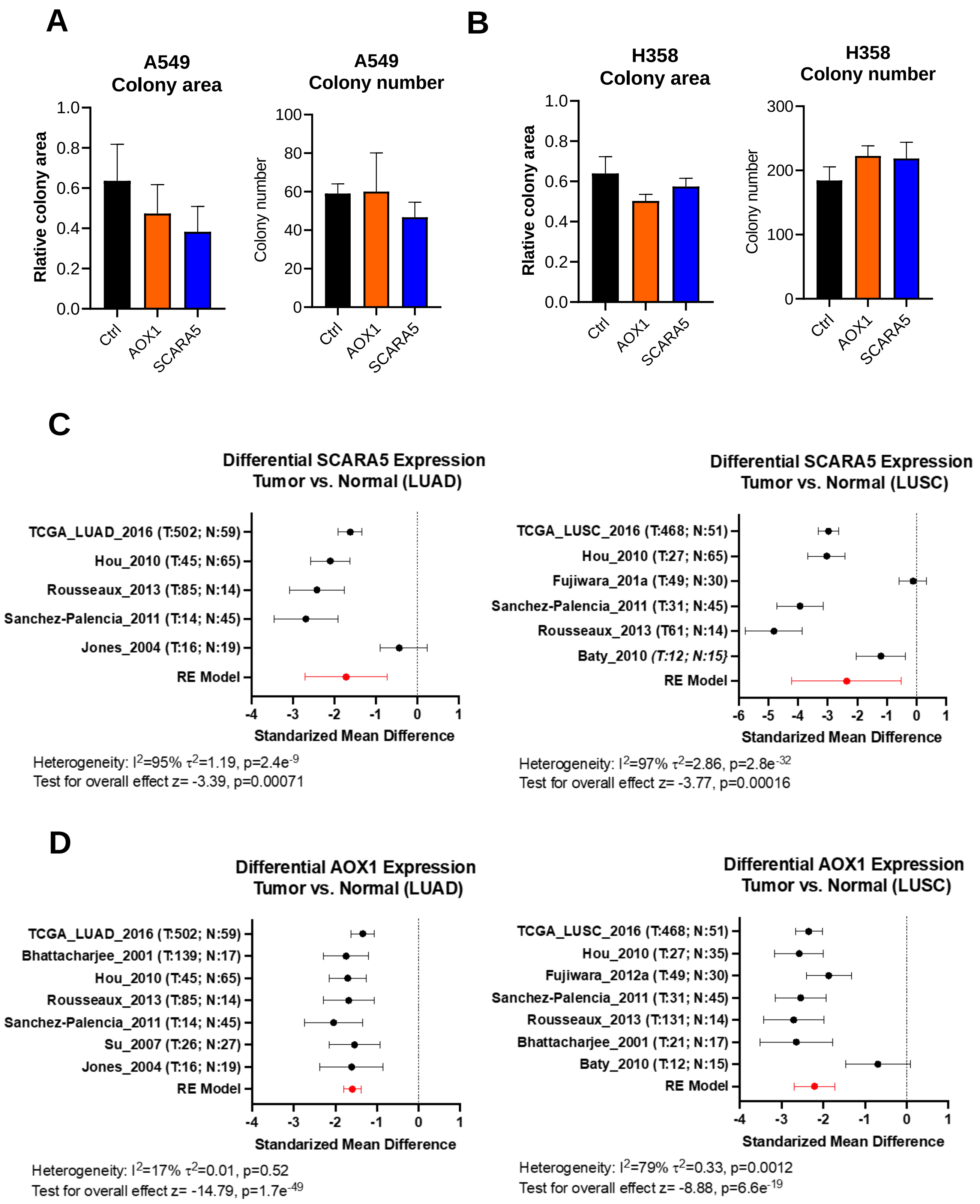
A
A549
Colony area
Rlative colony area
1.0
0.8
0.6
0.4
0.2
0.0
Ctrl
AOX1
SCARA5
A549
Colony number
Colony number
100
80
60
40
20
0
B
H358
Colony area
Rlative colony area
H358
Colony number
300
200
100
C
Differential SCARA5 Expression
Tumor vs. Normal (LUAD)
TCGA_LUAD_2016 (T:502; N:59)
Hou_2010 (T:45; N:65)
Rousseaux_2013 (T:85; N:14)
Sanchez-Palencia_2011 (T:14; N:45)
Jones_2004 (T:16; N:19)
RE Model
Standarized Mean Difference
Heterogeneity: I2=95% τ2=1.19, p=2.4e-9
Test for overall effect z= -3.39, p=0.00071
Differential SCARA5 Expression
Tumor vs. Normal (LUSC)
TCGA_LUSC_2016 (T:468; N:51)
Hou_2010 (T:27; N:65)
Fujiwara_201a (T:49; N:30)
Sanchez-Palencia_2011 (T:31; N:45)
Rousseaux_2013 (T61; N:14)
Baty_2010 (T:12; N:15)
RE Model
Standarized Mean Difference
Heterogeneity: I2=97% τ2=2.86, p=2.8e-32
Test for overall effect z= -3.77, p=0.00016
D
Differential AOX1 Expression
Tumor vs. Normal (LUAD)
TCGA_LUAD_2016 (T:502; N:59)
Bhattacharjee_2001 (T:139; N:17)
Hou_2010 (T:45; N:65)
Rousseaux_2013 (T:85; N:14)
Sanchez-Palencia_2011 (T:14; N:45)
Su_2007 (T:26; N:27)
Jones_2004 (T:16; N:19)
RE Model
Standarized Mean Difference
Heterogeneity: I2=17% τ2=0.01, p=0.52
Test for overall effect z= -14.79, p=1.7e-49
Differential AOX1 Expression
Tumor vs. Normal (LUSC)
TCGA_LUSC_2016 (T:468; N:51)
Hou_2010 (T:27; N:35)
Fujiwara_2012a (T:49; N:30)
Sanchez-Palencia_2011 (T:31; N:45)
Rousseaux_2013 (T:131; N:14)
Bhattacharjee_2001 (T:21; N:17)
Baty_2010 (T:12; N:15)
RE Model
Standarized Mean Difference
Heterogeneity: I2=79% τ2=0.33, p=0.0012
Test for overall effect z= -8.88, p=6.6e-19

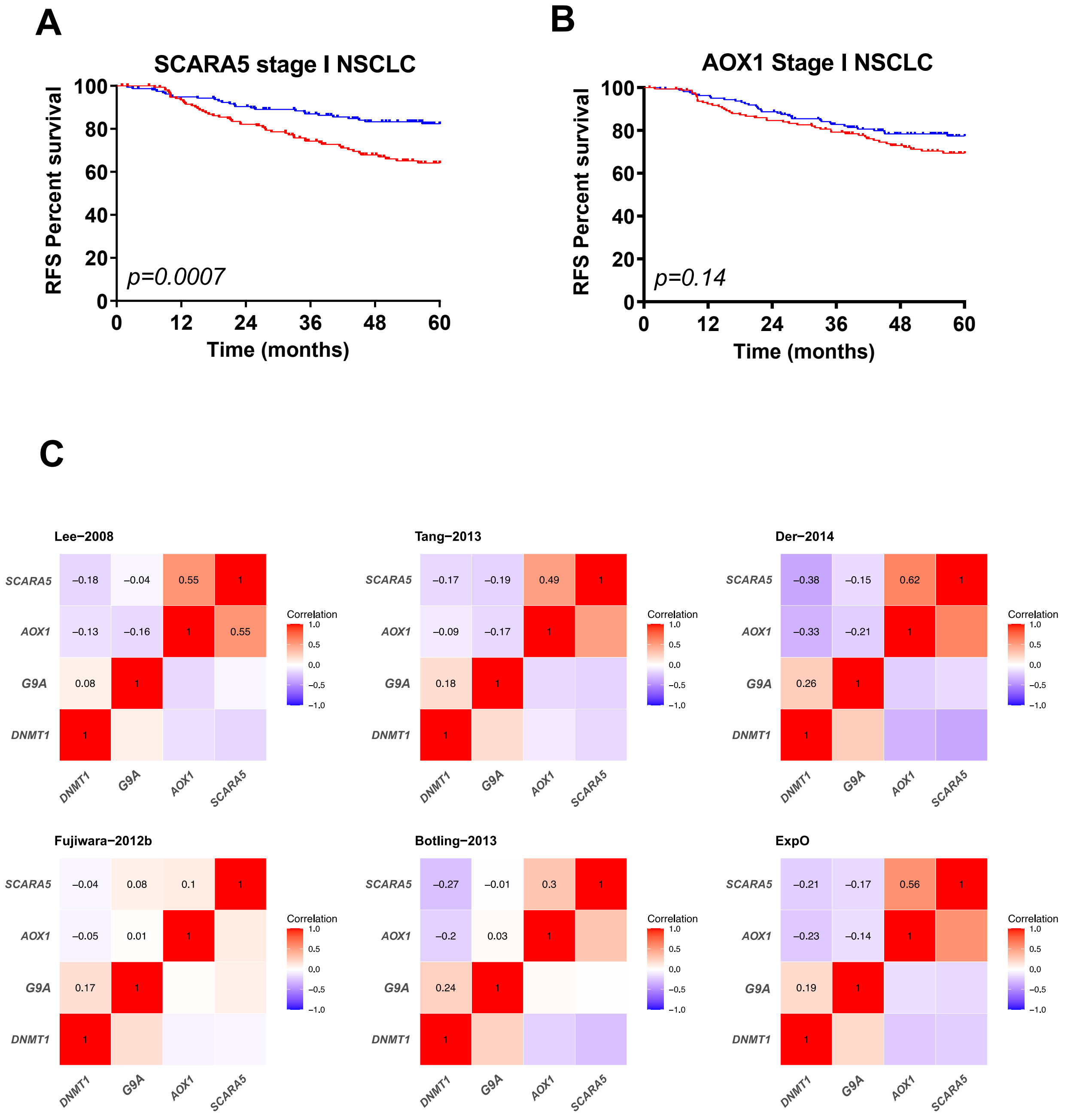
A
SCARA5 stage I NSCLC
RFS Percent survival
Time (months)
p=0.0007
B
AOX1 Stage I NSCLC
RFS Percent survival
Time (months)
p=0.14
C
Lee−2008
Tang−2013
Der−2014
Fujiwara−2012b
Botling−2013
ExpO
SCARA5
AOX1
G9A
DNMT1
Correlation

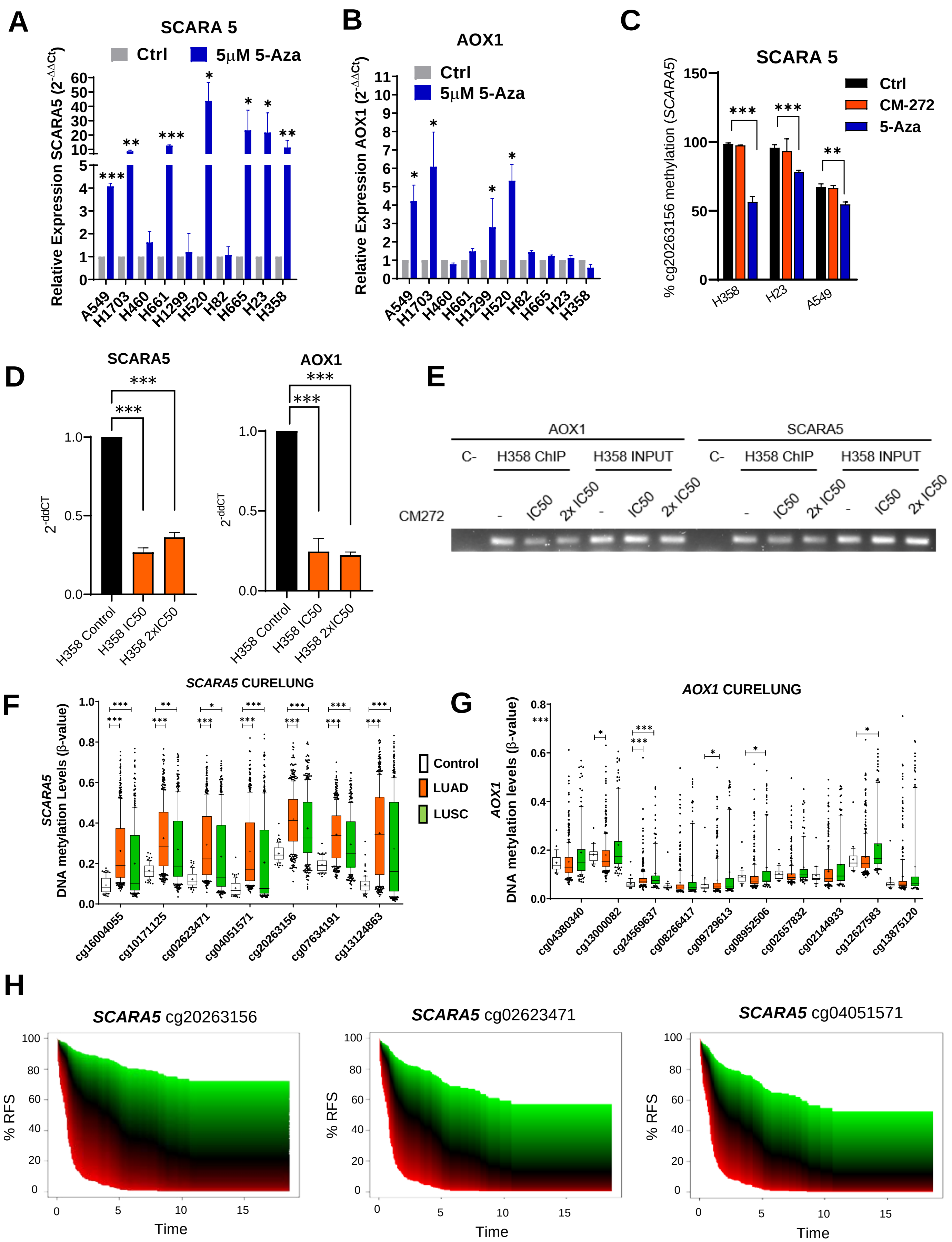
A
SCARA 5
Ctrl
5μM 5-Aza
Relative Expression SCARA5 (2^-ΔΔCt)
A549 H1703 H460 H661 H1299 H520 H82 H665 H23 H358
B
AOX1
Ctrl
5μM 5-Aza
Relative Expression AOX1 (2^-ΔΔCt)
A549 H1703 H460 H661 H1299 H520 H82 H665 H23 H358
C
SCARA 5
Ctrl
CM-272
5-Aza
% cg20263156 methylation (SCARA5)
H358
H23
A549
D
SCARA5
AOX1
2-ddCT
H358 Control
H358 IC50
H358 2xIC50
E
AOX1
SCARA5
C-
H358 ChIP
H358 INPUT
CM272
IC50
2x IC50
F
SCARA5 CURELUNG
SCARA5 DNA metylation Levels (β-value)
Control
LUAD
LUSC
cg16004055 cg10171125 cg02623471 cg04051571 cg20263156 cg07634191 cg13124863
G
AOX1 CURELUNG
AOX1 DNA metylation levels (β-value)
cg04380340 cg13000082 cg24569637 cg08266417 cg09729613 cg08952506 cg02657832 cg02144933 cg12627583 cg13875120
H
SCARA5 cg20263156
SCARA5 cg02623471
SCARA5 cg04051571
% RFS
Time

Supplementary Figure 10 *Exposito et al*

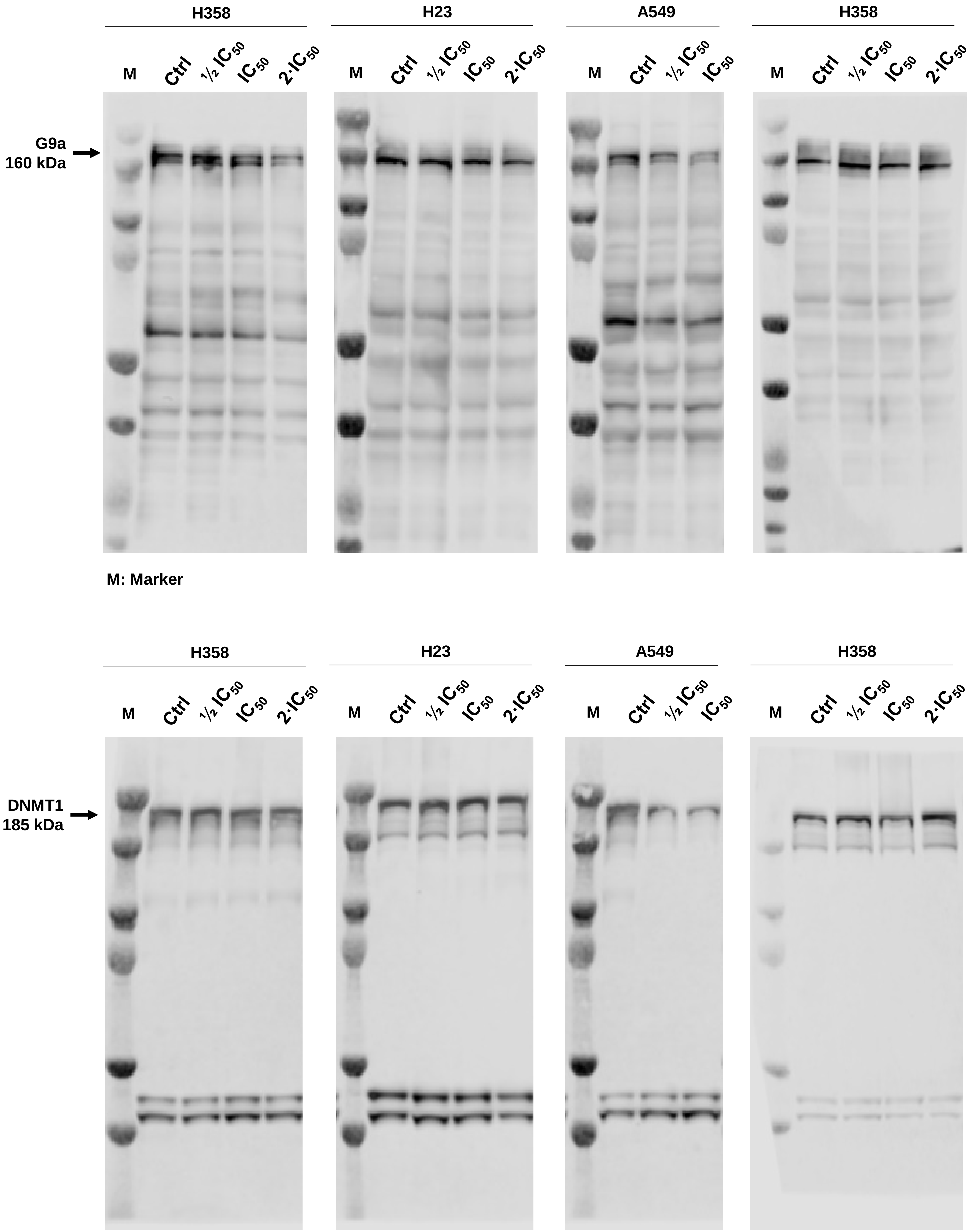

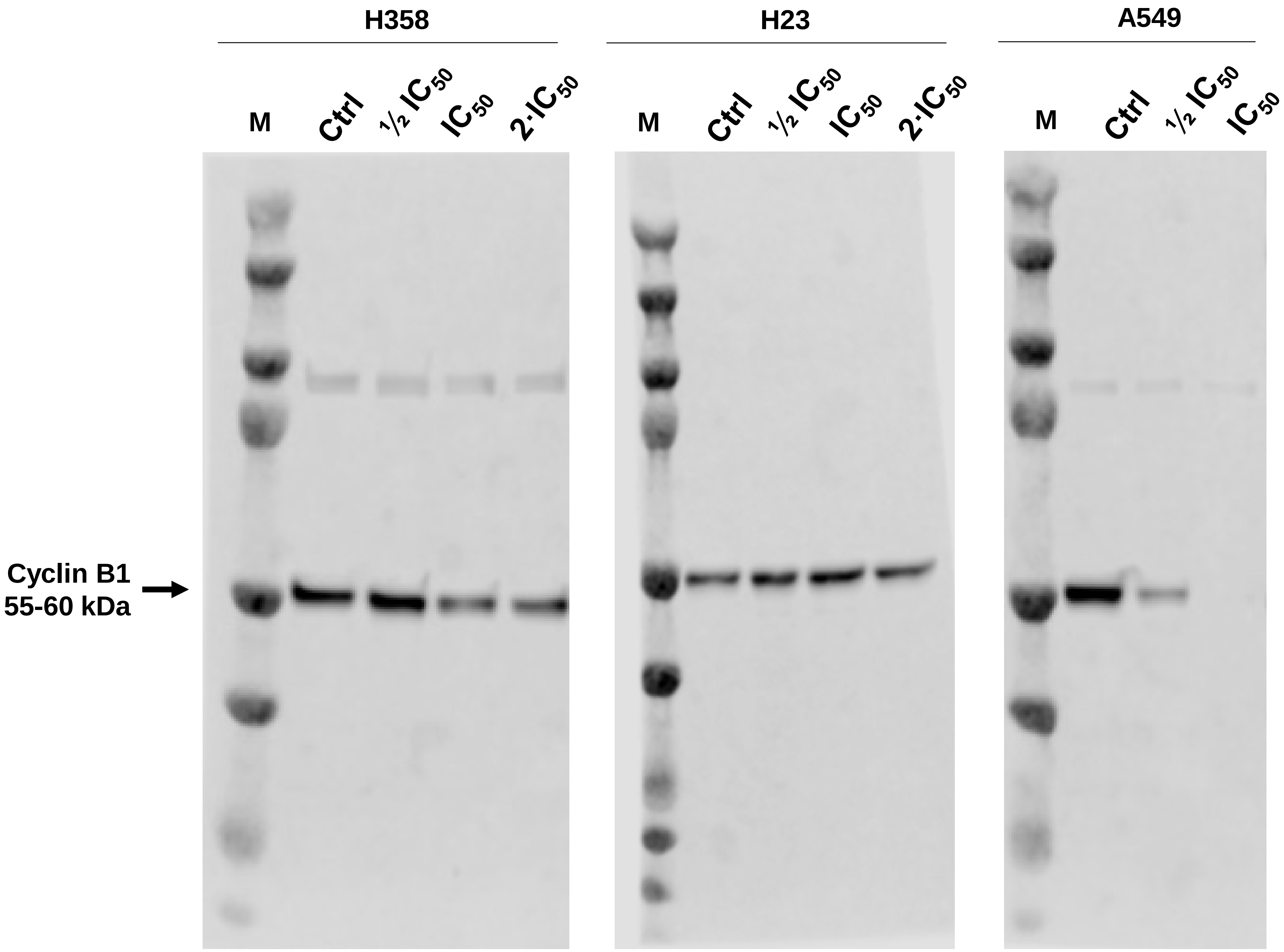

H358
H23
A549
M
Ctrl
½ IC50
IC50
2·IC50
Cyclin B1
55-60 kDa

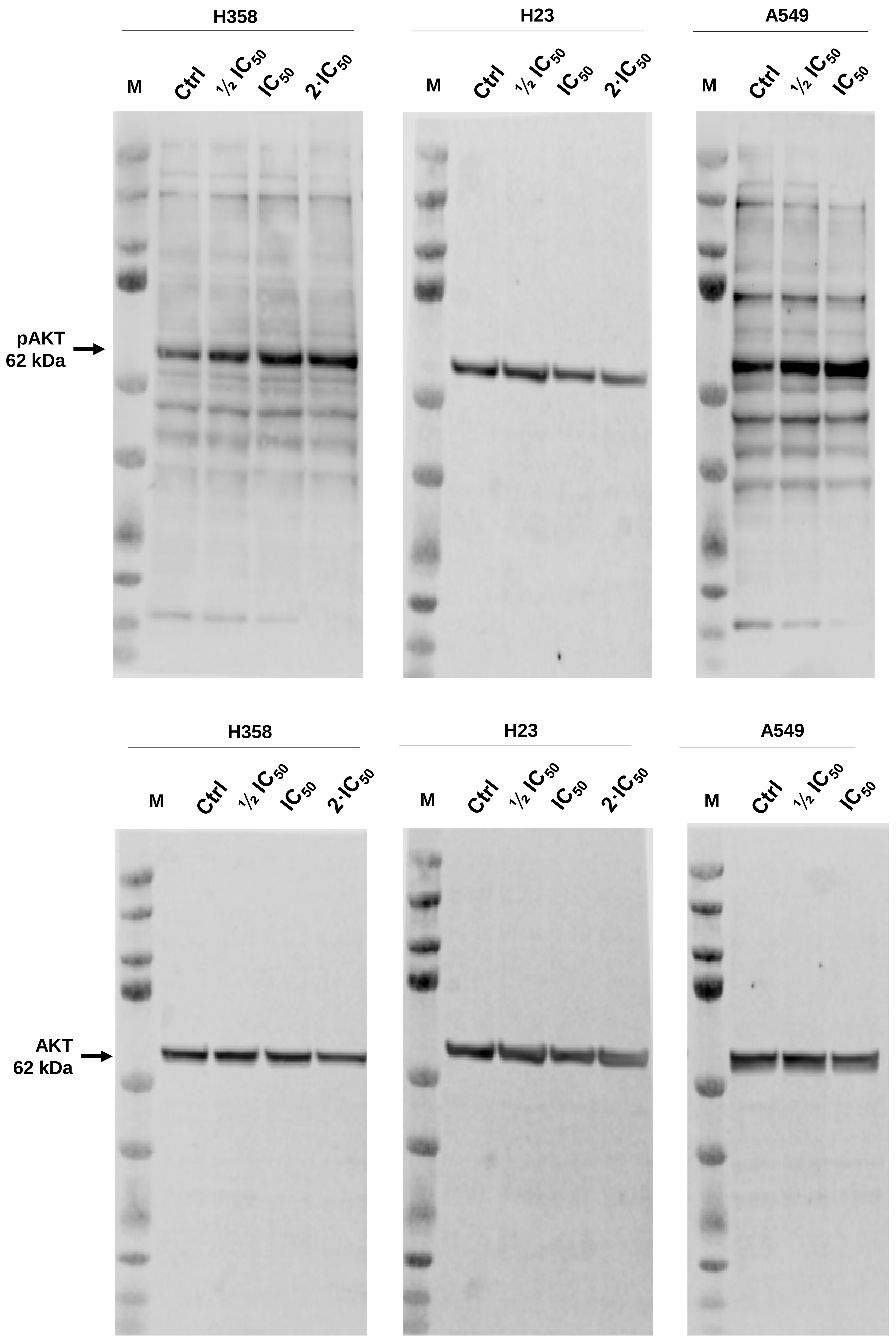
H358
H23
A549
M
Ctrl
½ IC50
IC50
2·IC50
pAKT
62 kDa
AKT
62 kDa

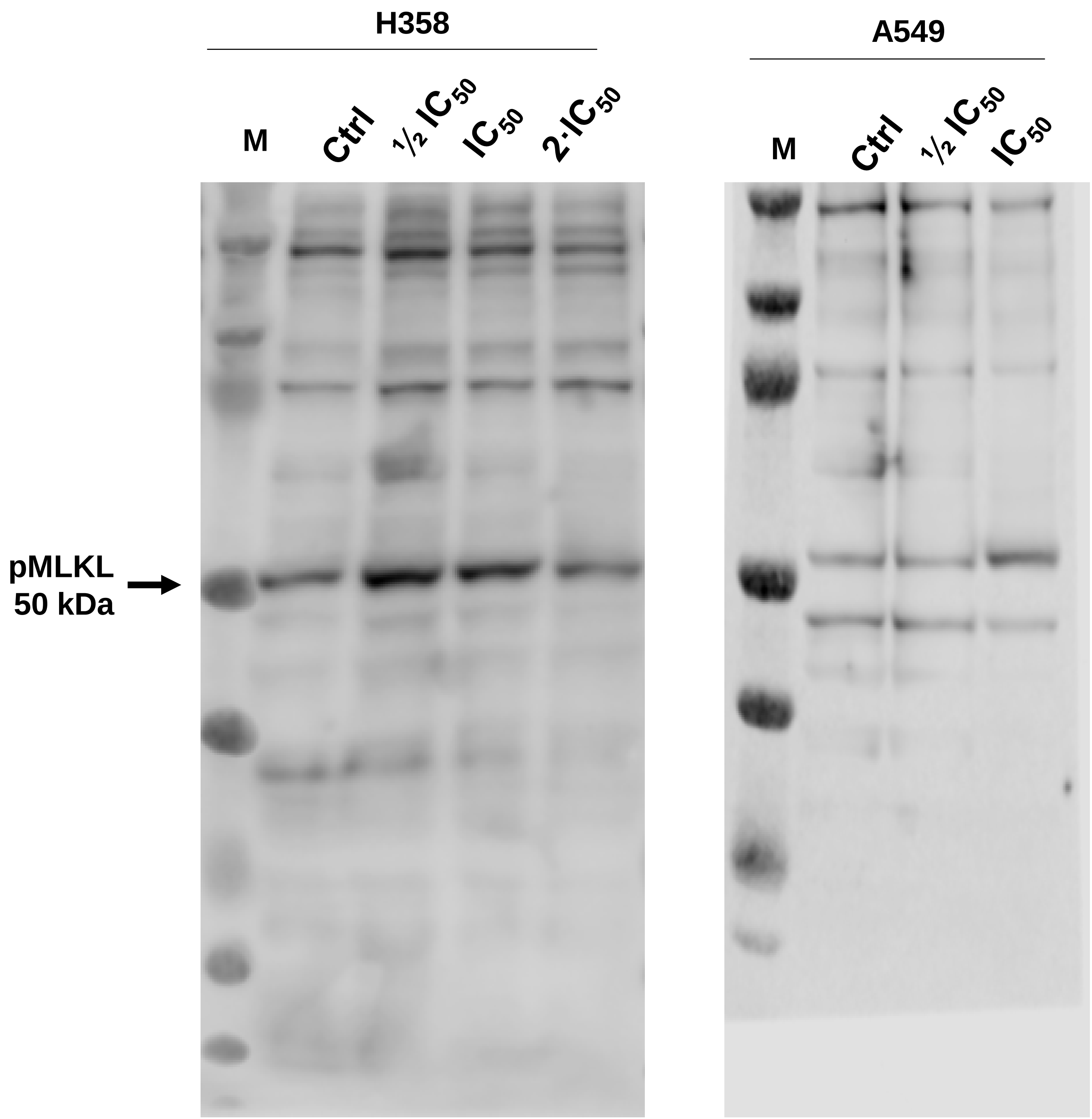
H358
A549
M
Ctrl
½ IC50
IC50
2·IC50
M
Ctrl
½ IC50
IC50
pMLKL
50 kDa
