# Supplementary figures and images for: G9a/DNMT1 co-targeting inhibits non-small cell lung cancer growth and reprograms tumor cells to respond to cancer-drugs through SCARA5 and AOX1
Source: Cell Death Dis. 2024 Nov 2;15(11):787. doi: 10.1038/s41419-024-07156-w (PMC11531574; doi:10.1038/s41419-024-07156-w)

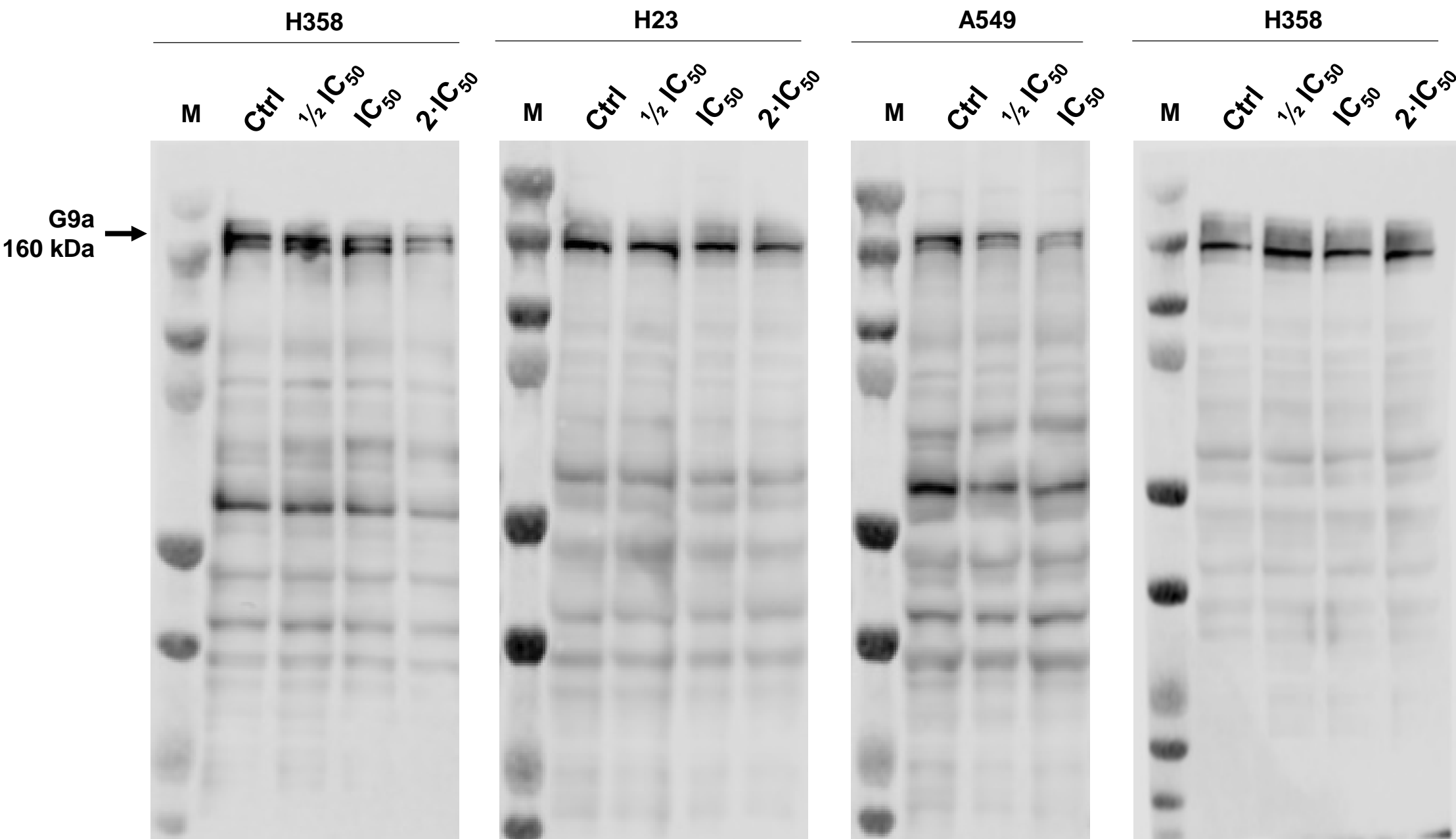

**M: Marker**

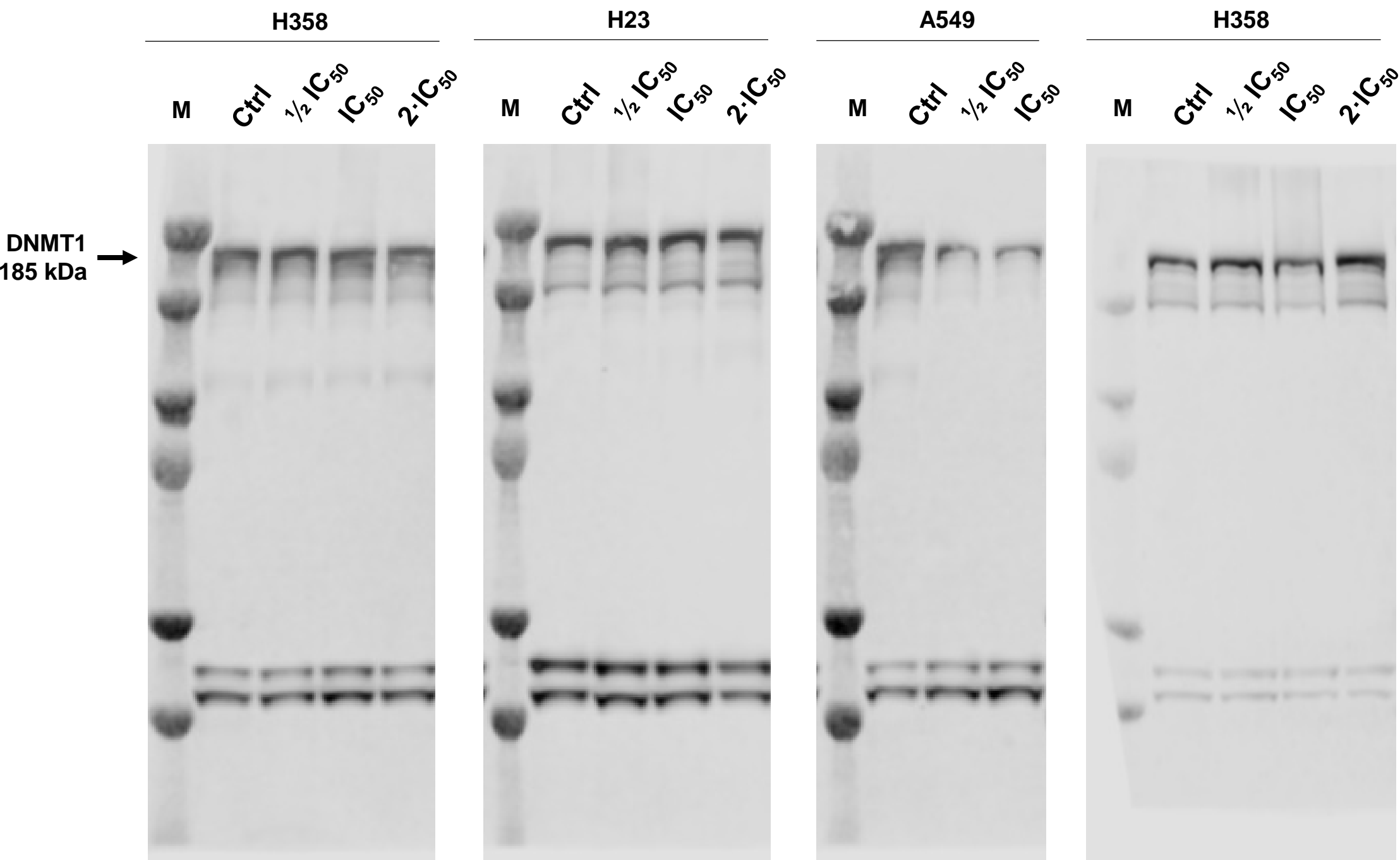

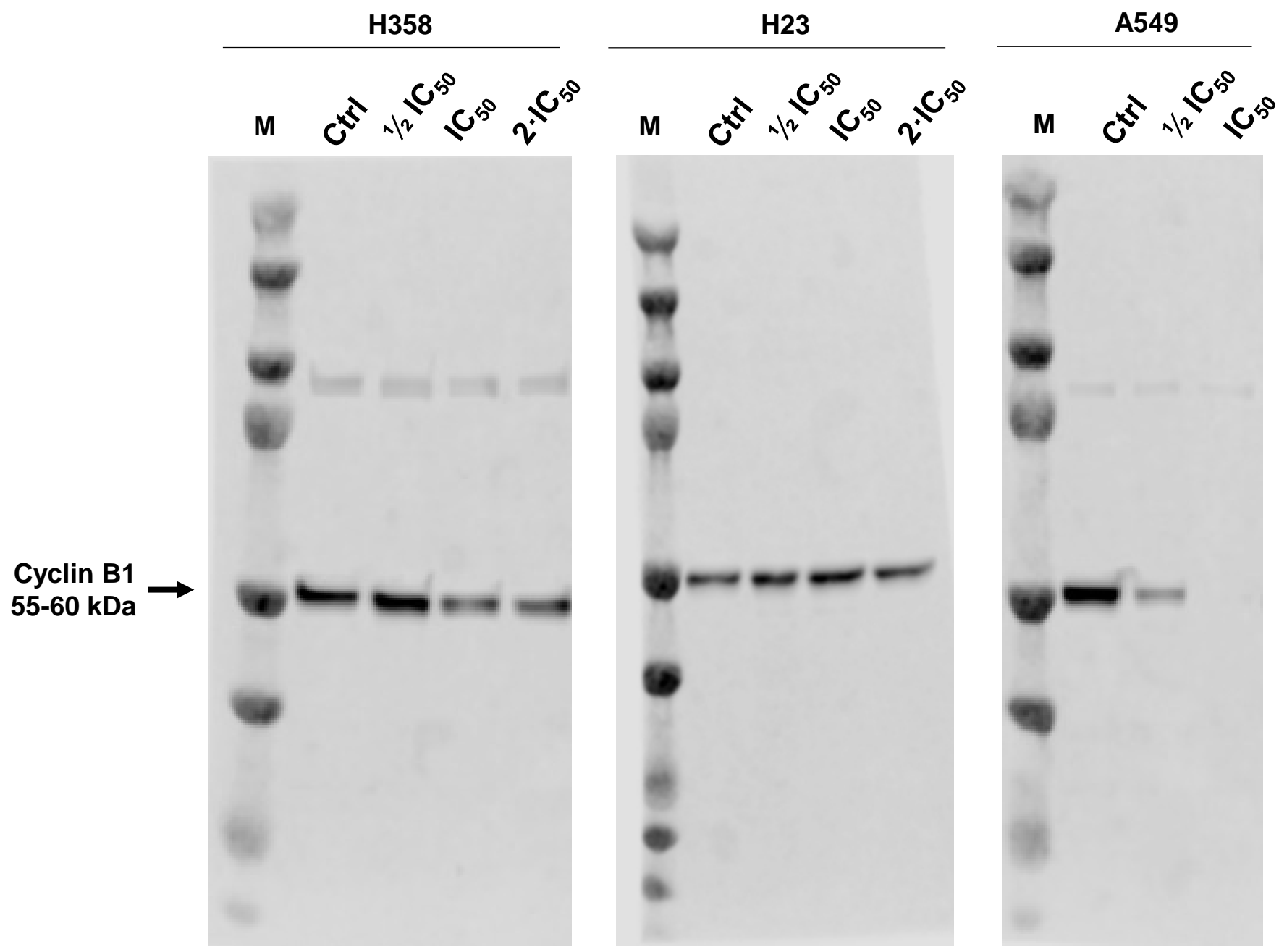

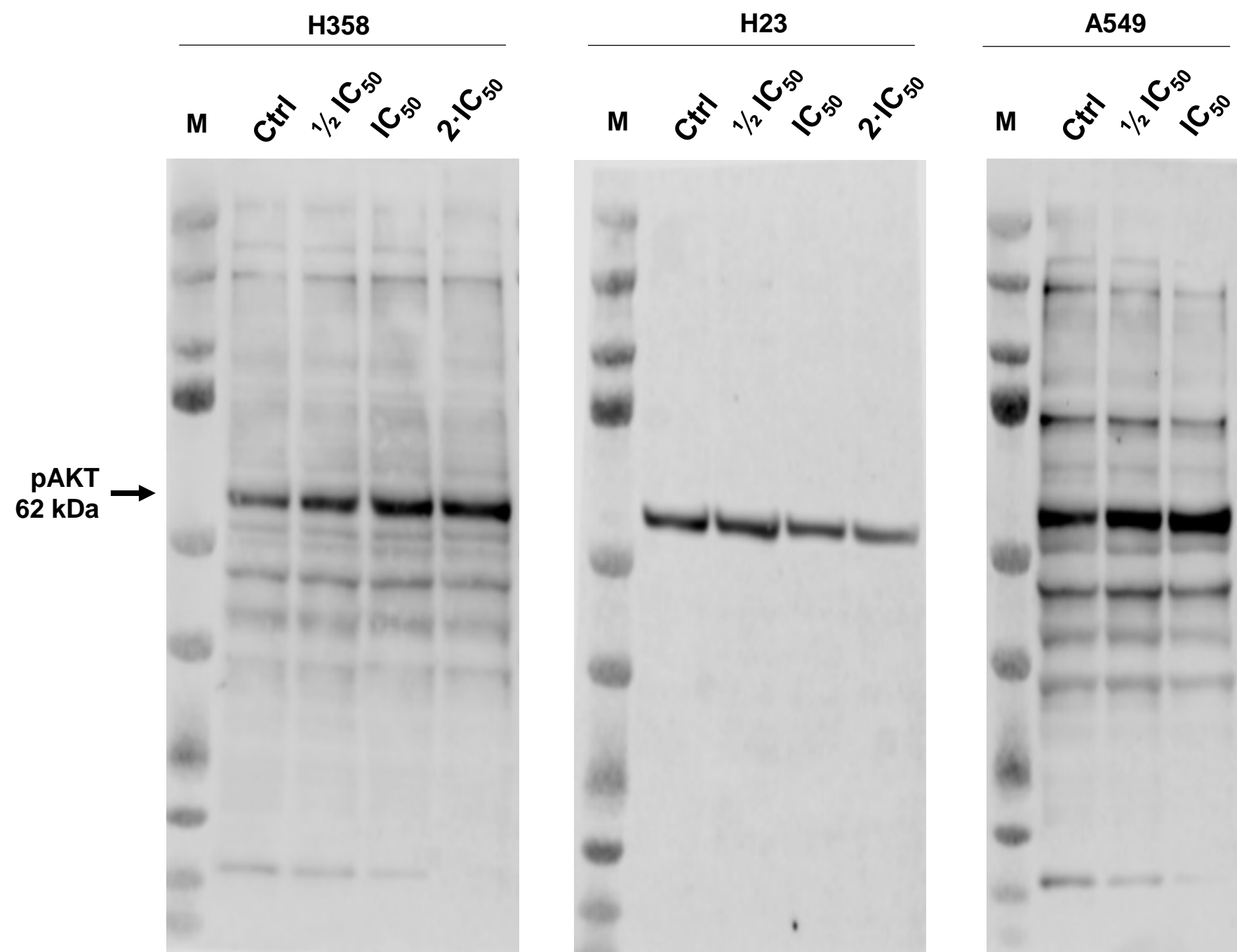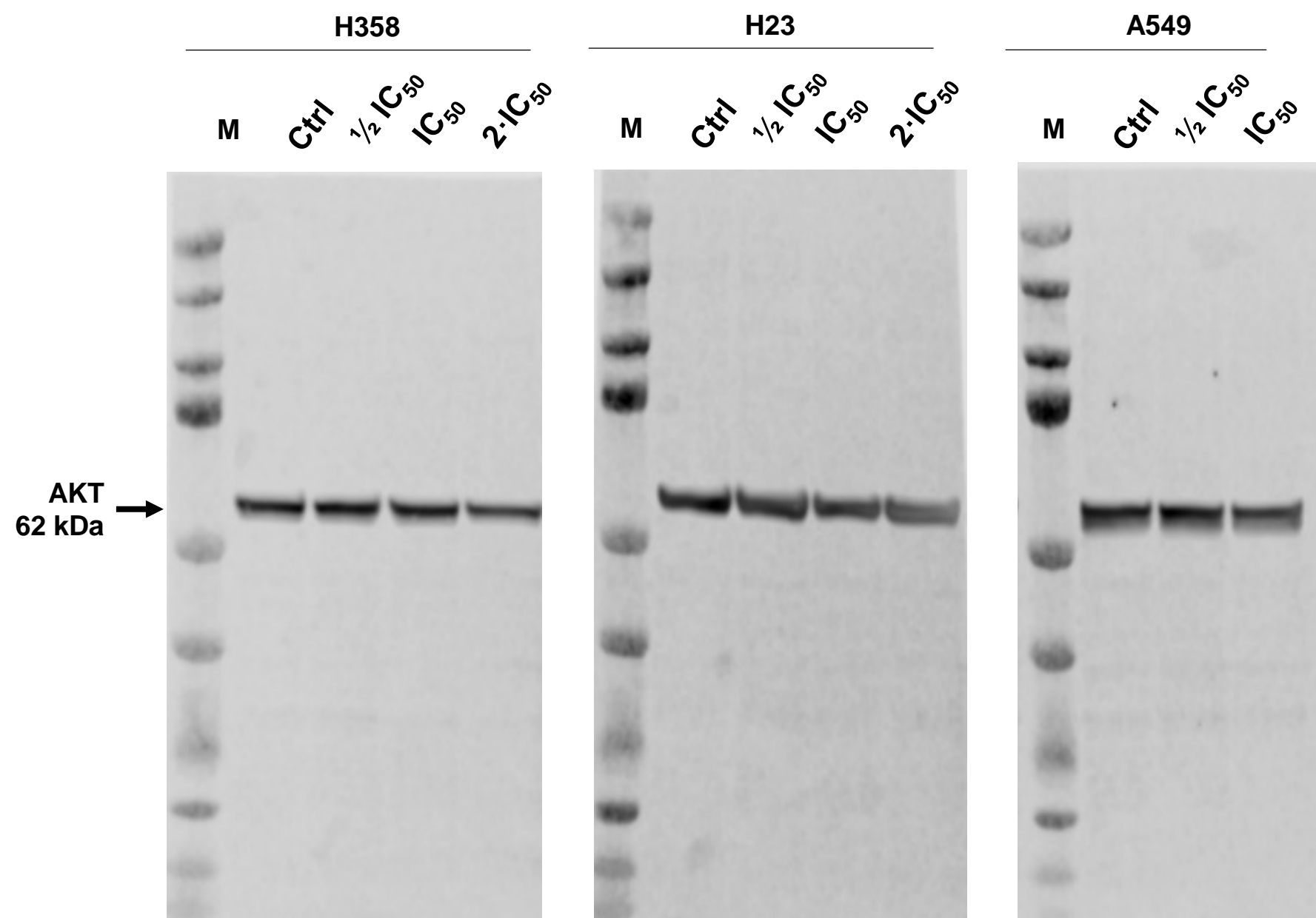

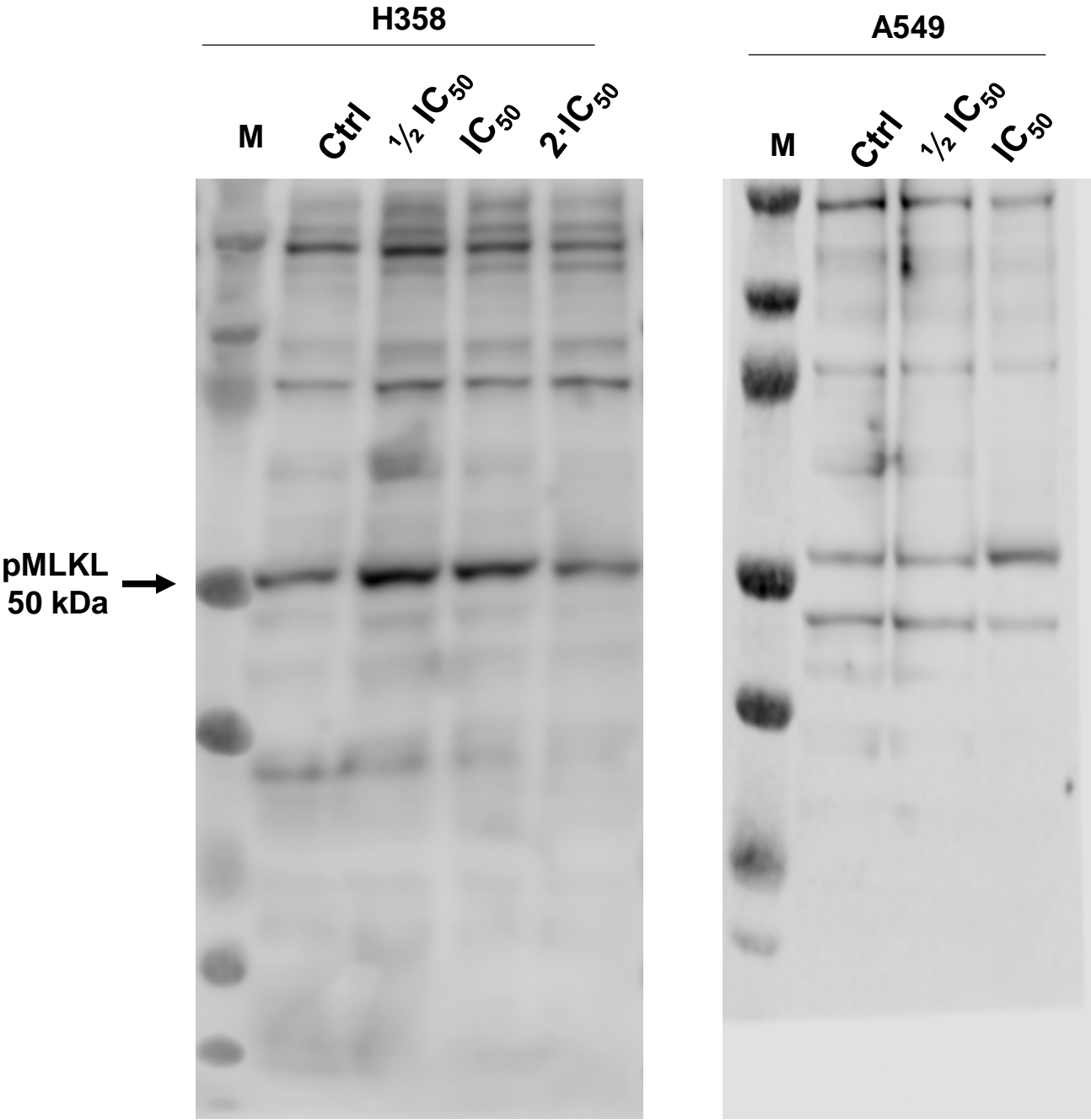

Supplement: Supplementary file 2 — Original Data [file 41419_2024_7156_MOESM2_ESM.pdf]
